# Estimating the Reach of a Manifold via its Convexity Defect Function

**DOI:** 10.1007/s00454-021-00290-8

**Published:** 2021-06-14

**Authors:** Clément Berenfeld, John Harvey, Marc Hoffmann, Krishnan Shankar

**Affiliations:** 1 Université Paris-Dauphine PSL, CEREMADE, Place du Maréchal de Lattre de Tassigny, 75016 Paris, France; 2 Department of Mathematics, Swansea University, Fabian Way, Swansea, SA1 8EN UK; 3 National Science Foundation, 2415 Eisenhower Avenue, Alexandria, VA 22314 USA

**Keywords:** Point clouds, Manifold reconstruction, Minimax estimation, Convexity defect function, Reach, 62C20, 62G05, 53A07, 53C40

## Abstract

The reach of a submanifold is a crucial regularity parameter for manifold learning and geometric inference from point clouds. This paper relates the reach of a submanifold to its convexity defect function. Using the stability properties of convexity defect functions, along with some new bounds and the recent submanifold estimator of Aamari and Levrard (Ann. Statist. **47**(1), 177–204 (2019)), an estimator for the reach is given. A uniform expected loss bound over a ${\mathscr {C}}^k$ model is found. Lower bounds for the minimax rate for estimating the reach over these models are also provided. The estimator almost achieves these rates in the ${\mathscr {C}}^3$ and ${\mathscr {C}}^4$ cases, with a gap given by a logarithmic factor.

## Introduction

### Motivation

The reach of a submanifold $\mathbf{M } \subseteq \mathbf{R } ^D$ is a geometric invariant which measures how tightly the submanifold folds in on itself. Dating back to Federer [12], it encodes both local curvature conditions as well as global ‘bottlenecks’ arising from two regions of the manifold that are far apart in the manifold’s intrinsic metric but are close in the ambient Euclidean metric. The reach is a key regularity parameter in the estimation of other geometric information. Methods and algorithms from topological data analysis often use the reach as a ‘tuning parameter’. The correctness of their results depends on setting this parameter correctly.

Statistical inference from point clouds has become an active area. In a probabilistic framework, a *reach condition*, meaning that the reach of the submanifold under study is bounded below, is usually necessary in order to obtain minimax inference results in manifold learning. These include: homology inference [7, 17], curvature [3], reach estimation itself [1], as well as manifold estimation [3, 13, 15]. In this context, there is a risk of algorithms being applied as ‘black boxes’ without attention to their underlying assumptions. Efficient reach estimation would be a vital addition to this field, providing a so-called sanity test of other results.

In this direction, the way was paved in [1]: under some specific assumptions, an estimator of the reach has been proposed and studied when the observation is an *n*-sample of a smooth probability distribution supported on an unknown *d*-dimensional submanifold $\mathbf{M }$ of a Euclidean space $\mathbf{R } ^D$ together with the tangent spaces at each sampled point. For certain types of ${\mathscr {C}}^3$-regularity models, the estimator, based on a representation of the reach in terms of points of $\mathbf{M }$ and its tangent spaces [12, Thm. 4.18] achieves the rate $n^{-2/(3d-1)}$. A lower bound for the minimax rate of convergence is given by $n^{-1/d}$. In the special case when the reach of $\mathbf{M }$ is attained at a bottleneck, the algorithm in [1] achieves this rate. However, in general, one does not know whether this condition is satisfied a priori.

In this paper, we continue the study of reach estimation by taking a completely different route: we use the relationship between the reach of a submanifold of $\mathbf{R } ^D$ and its *convexity defect function*. This function was introduced in [6] and measures how far a (bounded) subset $\mathbf{X } \subseteq \mathbf{R } ^D$ is from being convex at a given scale. It is a powerful geometric tool which has other applications such as manifold reconstruction, see the recent work by Divol [10]. By establishing certain new quantitative properties of the convexity defect function of a submanifold $\mathbf{M } \subseteq \mathbf{R } ^D$ that relate to both its curvature and bottleneck properties, we show that the convexity defect function can be used to compute the reach of a submanifold. From this we obtain a method which transforms an estimator of $\mathbf{M }$, along with information on its error, into a new estimator of the reach.

The recent results of Aamari and Levrard in [3] provide an estimator of $\mathbf{M }$ which is optimal, to within logarithmic terms. Transforming this into an estimator of the reach, we obtain new convergence results over general ${\mathscr {C}}^k$-regularity models ($k\geqslant 3$). These rates improve upon the previous work of [1]. By establishing lower bounds for the minimax rates of convergence, we prove that our results are optimal up to logarithmic terms in the cases $k=3$ and $k=4$.

### Main Results

We present here one of several possible definitions of the reach. Given a submanifold $\mathbf{M } \subseteq \mathbf{R } ^D$, consider its $\delta$-offset given by the open set $\mathbf{M } ^{\delta } \subseteq \mathbf{R } ^D$, where

$$\begin{aligned} \mathbf{M } ^{\delta } = \bigcup _{p\in \mathbf{M } } B_{\delta }(p). \end{aligned}$$

Here $B_{\delta }(p)$ denotes the open Euclidean ball centered at *p* with radius $\delta$. For small enough $\delta$ (a uniform choice for such $\delta$ exists in general only when $\mathbf{M }$ is compact), one has the property that for all $y\in \mathbf{M } ^{\delta } \setminus \mathbf{M }$, there is a unique straight line from *y* to a point in $\mathbf{M }$ realizing the distance from *y* to $\mathbf{M }$. In other words, the metric projection $\uppi :\mathbf{M } ^{\delta } \rightarrow \mathbf{M }$ is well defined.

#### Definition 1.1

(Federer [12]) The *reach* of a submanifold $\mathbf{M }$ is

$$\begin{aligned} \sup {\bigl \lbrace \delta \geqslant 0:\text {the nearest point projection }\uppi :\mathbf{M } ^{\delta } \rightarrow \mathbf{M } \text { is well defined} \bigr \rbrace }. \end{aligned}$$

We denote the reach by $R(\mathbf{M } )$ or simply *R* when the context is clear.

Other equivalent characterizations of the reach exist. For example, in Sect. 4.1 below, we use the characterization from [12, Thm. 4.18]. More recently, [8, Thm. 1] defined the reach in terms of the metric distortion.

Our main results are obtained for a statistical model which imposes certain standard regularity conditions on the manifolds being considered, requires that they are compact and connected, and also imposes conditions on the distributions being considered which have support on those manifolds. The set of distributions satisfying these constraints on ${\mathscr {C}}^k$ manifolds is denoted in the results below by ${\mathscr {P}}^k$ and these constraints are elaborated upon in Sects. 3 and 6.

#### Theorem 1.2

For *d*-dimensional submanifolds of regularity ${\mathscr {C}}^k$ with $k \geqslant 3$, and for sufficiently large *n*, there exists an estimator ${\widehat{R}}$, explicitly constructed in Sect. 6 below, that satisfies

$$\begin{aligned} \sup _{P \in {\mathscr {P}}^k} \mathbf{E } _{P^{\otimes n}}[| {\widehat{R}} - R|] \leqslant C{\left\{ \begin{array}{ll} \biggl ( \dfrac{\log n}{n-1} \biggr ) ^{\!1/d} &{} \text {for }k =3,\\ \biggl ( \dfrac{\log n}{n-1} \biggr ) ^{\!k/(2d)} &{}\text {for }k\geqslant 4, \end{array}\right. } \end{aligned}$$

where ${\widehat{R}}$ denotes an estimator of the reach $R = R(\mathbf{M } )$ constructed from an *n*-sample $(X_1,\ldots , X_n)$ of independent random variables with common distribution $P \in {\mathscr {P}}^k$. The quantity $C>0$ depends on *d*, *k*, and the regularity parameters that define the class ${\mathscr {P}}^k$, and the notation $\mathbf{E } _{P^{\otimes n}}[\,{\cdot }\,]$ refers to the expectation operator under the distribution $P^{\otimes n}$ of the *n*-sample $(X_1,\ldots , X_n)$.

We also provide a lower bound for the minimax convergence rate. In case $k=3,4$, our estimators are almost optimal, with a gap given by a $\log n$ factor.

#### Theorem 1.3

For certain values of the regularity parameters (depending only on *d* and *k*),

$$\begin{aligned} \inf _{\widehat{R}}\sup _{P \in {\mathscr {P}}^k} \mathbf{E } _{P^{\otimes n}} [| \widehat{R} -R|] \geqslant c n^{-(k-2)/d}, \end{aligned}$$

where the infimum is taken over all the estimators $\widehat{R} = \widehat{R}(X_1 ,\ldots ,X_n )$, and $c>0$ depends on *d*, *k*, and the regularity parameters.

These results are of an entirely theoretical nature. The question of practical implementation remains, although it is not of primary interest for the paper. Starting with a point cloud $X_1,\ldots , X_n$, one may implement the following protocol:

- Estimate $\mathbf{M }$ from the data $X_1,\ldots , X_n$ by the best available manifold reconstruction method ${\widehat{\mathbf{M } }}$, or, indeed, by any other method.
- Compute $h_{{\widehat{\mathbf{M } }}}$ (Definition 4.1) and derive ${\widehat{R}}$ thanks to Definition 6.7.

The only inputs are ${\widehat{\mathbf{M } }}$ and the regularity parameters that define the class ${\mathscr {P}}^k$. It is a common practice in statistics to assume some prior knowledge of the class in order to constrain the problem. However, the quantities $C_{d,k,R_{\min }}$ and *C* in Theorem 6.8 are unknown, which creates difficulties in deriving the accuracy of the estimator ${\widehat{R}}$ and, for example, calculating a confidence interval. This is common to every minimax result and could in practice be treated by estimating the variance of the estimator via any conventional computational method such as the bootstrap [11]. A more detailed discussion lies outside the scope of the present paper.

### Organization of the Paper

The paper is divided into two halves: a first half that is mainly geometric in flavor and a second half which employs mainly statistical techniques. To that end Sects. 2–4 describe the geometric setting of this paper in some detail, Sect. 5 discusses the approximation of the reach in a deterministic setting, while Sects. 6 and 7 are devoted to showing that the new algorithm proposed to estimate the reach achieves the rates stated in Theorem 1.2 and to the proof of the lower bound for the minimax rate stated in Theorem 1.3.

Section 2: We elaborate on the geometry of the reach. We recall a dichotomy due to Aamari et al. [1] in Theorem 2.1 and we study in particular the distinction between *global reach* or *weak feature size* in Definition 2.2 and the *local reach* in Definition 2.3, according to the terminology of [1]. This is not apparent in the classical Definition 1.1 of Federer.

Section 3: A geometrical framework is given for studying reach estimation. We describe precisely a class ${\mathfrak {C}}^k_{R_{\mathrm{min}}, \mathbf{L } }$ of submanifolds, following Aamari and Levrard [3]. Manifolds $\mathbf{M }$ in this class admit a local parametrization at all points $p\in \mathbf{M }$ by the tangent space $T_p\mathbf{M }$, which is the inverse of the projection to the tangent space and satisfies certain ${\mathscr {C}}^k$ bounds.

Section 4: This section is devoted to the study of the *convexity defect function* $h_\mathbf{M }$ of $\mathbf{M }$ as introduced in [6] and its properties. We show how the local reach can be calculated from the values of $h_\mathbf{M }$ near the origin in Proposition 4.3 and how the weak feature size (the global reach) appears as a discontinuity point of $h_\mathbf{M }$ whenever it is smaller than the local reach. This is done by proving an upper bound on $h_\mathbf{M }$ in Proposition 4.4. Propositions 4.3 and 4.4 are central to the results of the paper.

Section 5: When we attempt to estimate the reach in later sections, we will not know $\mathbf{M }$ exactly. Instead, we will know it up to some statistical error coming from an estimator. Propositions 5.1 and 5.3 give approximations of the local reach and the weak feature size, respectively, calculated from some proxy ${\widetilde{\mathbf{M } }}$. The errors of the approximations are given in terms of the Hausdorff distance ${{\,\mathrm{H}\,}}(\mathbf{M } ,{\widetilde{\mathbf{M } }})$.

Section 6: Building on the definitions in Sect. 3, a statistical framework is described within which we study reach estimation in a minimax setting. This defines a class ${\mathscr {P}}^k$ of admissible distributions *P* over their support $\mathbf{M }$, the submanifold of interest, which belongs to the class ${\mathfrak {C}}^k_{R_{\mathrm{min}}, \mathbf{L } }$. To apply the results of the previous section, we may use the Aamari–Levrard estimator [3] ${\widehat{\mathbf{M } }}$ of $\mathbf{M }$ from a sample $(X_1,\ldots , X_n)$ as the proxy ${\widetilde{\mathbf{M } }}$ for $\mathbf{M }$. This estimator is almost optimal over the class ${\mathscr {P}}^k$. This yields estimators of the local reach and finally of the reach $R(\mathbf{M } )$ in Sect. 6. We then prove the upper bounds announced in Theorem 1.2 above in Theorems 6.5–6.8.

Section 7: Using the classical Le Cam testing argument we obtain minimax lower bounds as announced in Theorem 1.3.

## Geometry of the reach

The reach of a submanifold $\mathbf{M }$, which we will denote by $R(\mathbf{M } )$, or simply *R*, is an unusual invariant. Definition 1.1 conceals what is almost a dichotomy—the reach of a submanifold can be realized in two very different ways. This is made precise by the following result.

### Theorem 2.1

([1, Thm. 3.4]) Let $\mathbf{M } \subseteq \mathbf{R } ^D$ be a compact submanifold with reach $R(\mathbf{M } ) > 0$. At least one of the following two assertions holds.

(Global case): $\mathbf{M }$ has a bottleneck, that is, there exist $q_1, q_2 \in \mathbf{M }$ such that $(q_1 + q_2)/2 \in {\text {Med}}(\mathbf{M } )$ and $\Vert q_1 - q_2\Vert = 2 R(\mathbf{M } )$.
(Local case): There exists $q_0 \in \mathbf{M }$ and an arc-length parametrized geodesic $\gamma$ such that $\gamma (0)=q_0$ and $\Vert \gamma ''(0) \Vert = 1/R(\mathbf{M } )$.

Here, ${\text {Med}}(\mathbf{M } )$ is the medial axis of $\mathbf{M }$, i.e., the subset of ${\mathbf{R }}^D$ on which the nearest point projection on $\mathbf{M }$ is ill defined, namely

$$\begin{aligned} {\text {Med}}(\mathbf{M } ) = \{z\in {\mathbf{R }}^D :\exists \, p,q \in \mathbf{M } ,\,p \ne q,\,d(z,p) = d(z,q) = d(z,\mathbf{M } )\}. \end{aligned}$$

We say that the result above is only ‘almost’ a dichotomy because it is possible for both conditions to hold simultaneously. The curve $\gamma$ could be one half of a circle with radius $R(\mathbf{M } )$ joining $q_1$ and $q_2$, for example, in which case the term ‘bottleneck’ might be considered a misnomer, or the points $q_1$ and $q_2$ might not lie on $\gamma$ at all, so that the two assertions hold completely independently.

This situation invites us to consider two separate invariants. One, the *weak feature size*, $R_{\mathrm{wfs}}$, is a widely studied invariant encoding large scale information such as bottlenecks. The second, which we will call the *local reach*, $R_{\ell }$, following [1], will encode curvature information. Theorem 2.1 states that the minimum of these two invariants is the reach,

$$\begin{aligned} R = \min {\lbrace R_{\ell }, R_{\mathrm{wfs}}\rbrace }. \end{aligned}$$

Note that, in Riemannian geometry, the local reach is referred to as the *focal radius of* $\mathbf{M }$, while the reach itself is often referred to as the *normal injectivity radius of* $\mathbf{M }$.

### The Weak Feature Size

The weak feature size is defined in terms of critical points of the distance function from $\mathbf{M }$ (in the sense of Grove and Shiohama; see for instance [14, p. 360]).

Consider the function, $d_{\mathbf{M } }:\mathbf{R } ^D \rightarrow \mathbf{R }$ defined by $d_{\mathbf{M } } (y) = \inf _{p \in \mathbf{M } } \Vert y - p\Vert .$ Note that $\mathbf{M } = d_{\mathbf{M } }^{-1}(0)$. Following [6], let $\Gamma _{\mathbf{M } }(y) = \{x \in \mathbf{M } : d_{\mathbf{M } }(y,\mathbf{M } ) = \Vert x - y \Vert \}$, i.e., those *x* in $\mathbf{M }$ realizing the distance between *y* and $\mathbf{M }$. Then we define a generalized gradient as

$$\begin{aligned} \nabla _{\mathbf{M } }(y) := \dfrac{y \hbox {-} \mathrm{Center}(\Gamma _{\mathbf{M } }(y))}{d_{\mathbf{M } }(y, \mathbf{M } )}, \end{aligned}$$

where $\hbox {Center}(\sigma )$ is defined as the center of the smallest (Euclidean) ball enclosing the bounded subset $\sigma \subseteq \mathbf{R } ^D$. This generalized gradient $\nabla _{\mathbf{M } }$ for $d_{\mathbf{M } }$ coincides with the usual gradient where $d_{\mathbf{M } }$ is differentiable. We say that a point $y \in \mathbf{R } ^D \setminus \mathbf{M }$ is a *critical point* of $d_{\mathbf{M } }$ if $\nabla _{\mathbf{M } }(y) = \mathbf{0 }$.

For example, if *y* is the midpoint of a chord the endpoints of which meet the submanifold perpendicularly, then from *y* there are two shortest paths to $\mathbf{M }$ which travel in opposite directions. It follows that *y* is a critical point.

#### Definition 2.2

Given a submanifold $\mathbf{M }$ of $\mathbf{R } ^D$ let ${\mathcal {C}}$ denote the set of critical points of the distance function $d_{\mathbf{M } }$. The *weak feature size*, denoted $R_{\mathrm{wfs}}(\mathbf{M} )$ or simply $R_{\mathrm{wfs}}$, is then defined as $R_{\mathrm{wfs}}:= \inf { \{ d_{\mathbf{M } }(y):y\in {\mathcal {C}}\}}$.

By Theorem 2.1, if the reach is realized globally then the first critical point will be the midpoint of a shortest chord which meets $\mathbf{M }$ perpendicularly at both ends, and so the weak feature size is equal to the reach.

### The Local Reach

In the local case, Theorem 2.1 tells us that the reach is determined by the maximum value of $\Vert \gamma '' \Vert$ over all arc-length parametrized geodesics $\gamma$. This can be formulated more concisely by considering instead the *second fundamental form*, $\mathrm{I\,\!I}$, which measures how the submanifold $\mathbf{M }$ curves in the ambient Euclidean space $\mathbf{R } ^D$. We refer the reader to a standard text in Riemannian geometry such as [9] for a precise definition of the second fundamental form. Informally, the second fundamental form is defined as follows. For a pair of vector fields tangent to $\mathbf{M }$, the (Euclidean) derivative of one with respect to the other is not usually tangent to $\mathbf{M }$. In fact, the tangential component is the Levi-Civita connection of the induced (Riemannian) metric on $\mathbf{M }$. The normal, or perpendicular, component yields a symmetric, bilinear form, namely, the second fundamental form, denoted by $\mathrm{I\,\!I}_p$. In particular, if the norm of $\mathrm{I\,\!I}_p$ is small then $\mathbf{M }$ is nearly flat near *p* and if the norm is large then it is an area of high curvature.

#### Definition 2.3

Given a submanifold $\mathbf{M }$ of $\mathbf{R } ^D$ let $\mathrm{I\,\!I}_p$ denote the second fundamental form at $p \in \mathbf{M }$. The *local reach* of $\mathbf{M }$, denoted $R_\ell (\mathbf{M} )$ or simply $R_\ell$, is the quantity

$$\begin{aligned} R_{\ell } = \inf _{p \in \mathbf{M } }{ \biggl \{ \dfrac{1}{\Vert \mathrm{I\,\!I}_p\Vert _{\text {op}}} \biggr \}}. \end{aligned}$$

We use the term ‘local reach’ here to reflect the fact that this quantity is generated entirely by the local geometry. In differential geometry literature the local reach is referred to as the *focal radius* of the submanifold.

## Geometrical Framework

We define a class of manifolds which are suitable for the task of reach estimation. This class is the same as that considered by Aamari and Levrard [3] for other problems in minimax geometric inference. The class is that of ${\mathscr {C}}^k$ submanifolds, but with some additional regularity requirements. These guarantee the existence of a Taylor expansion of the embedding of the submanifold with bounded coefficients, as well as a uniform lower bound on the reach.

### Definition 3.1

(see [3])   For two fixed natural numbers $d < D$ and for some $k \geqslant 3$, $R_{\mathrm{min}}> 0$, and $\mathbf{L } = (L_{\perp }, L_3, \ldots , L_k)$, we let ${\mathfrak {C}}^k_{R_{\mathrm{min}}, \mathbf{L } }$ denote the set of *d*-dimensional, compact, connected submanifolds $\mathbf{M }$ of $\mathbf{R } ^D$ such that:

(i) $$R(\mathbf{M } ) \geqslant R_{\mathrm{min}}$$;
(ii) for all $p \in \mathbf{M }$, there exists a local one-to-one parametrization $\psi _p$ of the form
  $$\begin{aligned} \psi _p :B_{T_p \mathbf{M } } (0,r) \subseteq T_p \mathbf{M } \rightarrow \mathbf{M } ,\qquad v \mapsto p + v + \mathbf{N }_p(v), \end{aligned}$$
  for some $r \geqslant {1}/({4 L_{\perp }})$, with $\mathbf{N }_p \in {\mathscr {C}}^k (B_{T_p \mathbf{M } } (0,r), \mathbf{R } ^D)$ such that
  $$\begin{aligned} \mathbf{N }_p(0) = 0, \qquad \mathrm{d}_0 \mathbf{N }_p = 0, \qquad \Vert \mathrm{d}^2_v \mathbf{N }_p \Vert _{\text {op}} \leqslant L_{\perp }, \end{aligned}$$
  for all $$\Vert v \Vert \leqslant {1}/({4L_{\perp }})$$;
(iii) the differentials $\mathrm{d}^i_v \mathbf{N }_p$ satisfy $\Vert \mathrm{d}^i_v \mathbf{N }_p\Vert _{\text {op}} \leqslant L_i$ for all $3 \leqslant i \leqslant k$ and $\Vert v\Vert \leqslant {1}/({4L_{\perp }})$.

We define subclasses of ${\mathfrak {C}}^k_{R_{\mathrm{min}}, \mathbf{L } }$ as follows, using the gap $R_{\ell } - R_{\text {wfs}}$ between the weak feature size and the local reach. For fixed values of $R_{\min }$ and $\mathbf{L }$, we define

$$\begin{aligned} {\mathscr {M}}^k_0&= \bigl \{\mathbf{M } \in {\mathfrak {C}}^k_{R_{\mathrm{min}}, \mathbf{L } }:R_{\text {wfs}}(\mathbf{M } ) \geqslant R_{\ell }(\mathbf{M } ) \bigr \}\qquad \text {and}\\ {\mathscr {M}}^k_\alpha&= \bigl \{\mathbf{M } \in {\mathfrak {C}}^k_{R_{\mathrm{min}}, \mathbf{L } }:R_{\text {wfs}}(\mathbf{M } ) \leqslant R_{\ell }(\mathbf{M } ) - \alpha \bigr \},\quad \;\;\alpha >0. \end{aligned}$$

Note that

$$\begin{aligned} {\mathfrak {C}}^k_{R_{\mathrm{min}}, \mathbf{L } }= \bigcup _{\alpha \geqslant 0} {\mathscr {M}}^k_\alpha . \end{aligned}$$

Manifolds in ${\mathfrak {C}}^k_{R_{\mathrm{min}}, \mathbf{L } }$ admit a second parametrization, one that represents the manifold locally as the graph of a function over the tangent space so that the first non-zero term in the Taylor expansion is of degree two and is given by the second fundamental form. These parametrizations in general satisfy weaker bounds than $\mathbf{L }$. The degree *k* Taylor polynomial then gives an algebraic approximation of the manifold, which will be very useful in later calculations. The following lemma from [3] describes the Taylor expansion of a local parametrization at every point $p\in \mathbf{M }$.

### Lemma 3.2

([3, Lem. 2])   Let $k\geqslant 3$, $\mathbf{M } \in {\mathfrak {C}}^k_{R_{\mathrm{min}}, \mathbf{L } }$, and $r=\min {\lbrace R_{\mathrm{min}}, L_{\perp }^{-1}\rbrace }/4$. Then for all $p \in \mathbf{M }$ there is a local one-to-one parametrization around *p*, $\Phi _p :U \rightarrow \mathbf{M }$, for some $U \subset T_p \mathbf{M }$, which contains $B(p,r) \cap \mathbf{M }$ in its image, satisfies ${{\,\mathrm{pr}\,}}_{T_p \mathbf{M } } \circ \Phi _p (v) = v$ on its domain, and takes the form

$$\begin{aligned} \Phi _p(v)= & {} p + v + \dfrac{1}{2} T_2(v^{\otimes 2}) + \dfrac{1}{6} T_3(v^{\otimes 3})+\cdots \quad +\dfrac{1}{(k-1)!} T_{k-1}(v^{\otimes (k-1)})+{\mathcal {R}}_k(v), \end{aligned}$$

where $\Vert {\mathcal {R}}_k(v)\Vert \leqslant C \Vert v\Vert ^k$. Furthermore $T_2 = \mathrm{I\,\!I}_p$ and $\Vert T_i\Vert _{{{\,\mathrm{op}\,}}} \leqslant L'_i$, where $L'_i$ and *C* depend on *d*, *k*, $R_{\mathrm{min}}$, and $\mathbf{L }$, and the terms $T_2,\ldots , T_{k-1}, {\mathcal {R}}_k$ are all normal to $T_p \mathbf{M }$.

### Definition 3.3

We call the degree *j* truncation of the parametrization $\Phi _p$ given in Lemma 3.2 the *approximation of degree* *j* *to* $\mathbf{M }$ *around* *p* and write it as

$$\begin{aligned} \Phi _p^j(v) = p + v + \dfrac{1}{2} T_2(v^{\otimes 2}) + \dfrac{1}{6} T_3(v^{\otimes 3})+\ldots +\dfrac{1}{j!} T_j(v^{\otimes j}). \end{aligned}$$

## Convexity Defect Functions

The convexity defect function, originally introduced by Attali et al. [6], measures how far a subset $\mathbf{X } \subseteq \mathbf{R } ^D$ is from being convex at scale *t*. The goal of this section is to establish a relationship between the convexity defect function and the reach. The definition is valid for any compact subset of $\mathbf{R } ^D$. In this section we will principally consider the case of a closed submanifold $\mathbf{M }$ as before, but in the sequel we will need to know that this function can be defined in greater generality.

We recall the definition. Given a compact subset $\sigma \subseteq \mathbf{X }$, it is contained in a smallest enclosing closed ball in $\mathbf{R } ^D$. We define ${{\,\mathrm{Rad}\,}}\sigma$ to be the radius of this ball. We denote by ${{\,\mathrm{Hull}\,}}\sigma$ the convex hull of $\sigma$ in $\mathbf{R } ^D$. Then we define the *convex hull of* $\mathbf{X }$ *at scale* *t* to be the following subset of $\mathbf{R } ^D$:

$$\begin{aligned} {{\,\mathrm{Hull}\,}}(\mathbf{X } , t)\, =\! \bigcup _{\begin{array}{c} \sigma \subseteq \mathbf{X } \\ {{\,\mathrm{Rad}\,}}\sigma \leqslant t \end{array}} \!\!{{\,\mathrm{Hull}\,}}\sigma . \end{aligned}$$

For two compact subsets *A* and *B* of $\mathbf{R }^D$, we define the asymmetric distance ${{\,\mathrm{H}\,}}(A|B) = \sup _{a \in A} d(a,B)$ so that ${{\,\mathrm{H}\,}}(A,B) = \max {\lbrace {{\,\mathrm{H}\,}}(A|B), {{\,\mathrm{H}\,}}(B|A)\rbrace }$ is the symmetric Hausdorff distance.

### Definition 4.1

Given a compact subset $\mathbf{X } \subseteq \mathbf{R } ^D$, we define the *convexity defect function* $h_{\mathbf{X } }:\mathbf{R } _{\geqslant 0}\rightarrow \mathbf{R } _{\geqslant 0}$ by $h_{\mathbf{X } }(t) = {{\,\mathrm{H}\,}}({{\,\mathrm{Hull}\,}}(\mathbf{X } , t) , \mathbf{X } ) = {{\,\mathrm{H}\,}}({{\,\mathrm{Hull}\,}}(\mathbf{X } ,t)\,|\,\mathbf{X } )$.

**Fig. 1:**
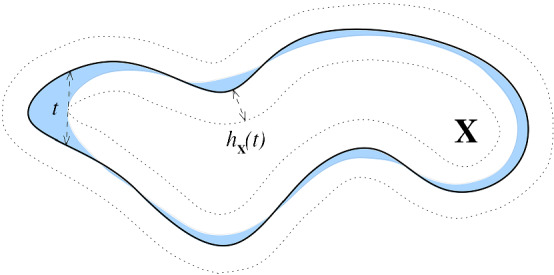
The convex hull at scale *t*, ${{\,\mathrm{Hull}\,}}(\mathbf{X } ,t)$ (in blue), of a curve $\mathbf{X }$ (in black). Enclosed between the dotted curves is the minimal tubular neighborhood around $\mathbf{X }$ that contains ${{\,\mathrm{Hull}\,}}(\mathbf{X } ,t)$ — its width is the convexity defect function $h_\mathbf{X } (t)$

We recall here from [6] some useful properties of $h_{\mathbf{X } }$ (Fig. 1).

1. $$h_{\mathbf{X } }(0) = 0$$.
2. $h_{\mathbf{X } }$ is non-decreasing on the interval $[0, {{\,\mathrm{Rad}\,}}\mathbf{X } ]$ and constant thereafter.
3. If ${\widetilde{\mathbf{X } }}\subseteq \mathbf{R } ^D$ satisfies H$(\mathbf{X } , {\widetilde{\mathbf{X } }}) < \varepsilon$, where *H* is the Hausdorff distance, then $h_{{\widetilde{\mathbf{X } }}}(t-\varepsilon ) - 2\varepsilon \leqslant h_{\mathbf{X } }(t) \leqslant h_{{\widetilde{\mathbf{X } }}}(t+\varepsilon ) + 2\varepsilon$ for any $t\geqslant \varepsilon$.
4. $h_{\mathbf{X } }(t) \leqslant t$ for all $t \geqslant 0$. Moreover, $h_{\mathbf{X } }(t_0) = t_0$ if and only if $t_0$ is a critical value of the distance function, $d_{\mathbf{X } }$.
5. If the reach, $R = R(\mathbf{X } ) >0$, then on [0, *R*) the function $h_{\mathbf{X } }(t)$ is bounded above by a quarter-circle of radius *R* centered on (0, *R*). In other words, $h_{\mathbf{X } }(t) \leqslant R - \sqrt{R^2 - t^2}$ for $t \in [0,R)$.

From item .4 and the definition of the weak feature size in terms of critical points of the distance function, the following proposition is immediate.

### Proposition 4.2

If $\mathbf{M }$ is a submanifold of $\mathbf{R } ^D$ then $R_{\mathrm{wfs}}= \inf { {\lbrace t >0: h_{\mathbf{M } }(t)=t\rbrace }}$.

We can also relate the local reach to the convexity defect function with the following proposition, which we will prove in Sect. 4.2.

### Proposition 4.3

Let $k \geqslant 4$. There exists a constant *C* (depending on $R_{\mathrm{min}}$, $\mathbf{L }$, *d*, and *k*) such that, for any sufficiently small non-negative real *t*, $t \leqslant t_{R_{\mathrm{min}}, \mathbf{L } ,d,k}$, and any $\mathbf{M } \in {\mathfrak {C}}^k_{R_{\mathrm{min}}, \mathbf{L } }$, we have

$$\begin{aligned} \biggl |h_{\mathbf{M } }(t) - \dfrac{t^2}{2R_\ell } \biggr | \leqslant Ct^4. \end{aligned}$$

In case $k=3$, there exists a constant $C'$ (depending on $R_{\mathrm{min}}, {\mathbf{L}}, d$) such that, for any sufficiently small non-negative real *t*, $t \leqslant t_{R_{\mathrm{min}}, \mathbf{L } ,d}$, and any $\mathbf{M } \in {\mathfrak {C}}^k_{R_{\mathrm{min}}, \mathbf{L } }$, we have

$$\begin{aligned} \biggl |h_{\mathbf{M } }(t) - \dfrac{t^2}{2 R_\ell } \biggr | \leqslant C't^3. \end{aligned}$$

We will write, somewhat informally,

$$\begin{aligned} R_{\ell } = \dfrac{1}{h''_{\mathbf{M } }(0)}. \end{aligned}$$

The function $h_{\mathbf{M } }$ is not actually twice differentiable; $h''_{\mathbf{M } }(0)$ here is a ‘pointwise second derivative’. Since $R = \min {\lbrace R_{\ell }, R_{\mathrm{wfs}}\rbrace }$, these two propositions show how the convexity defect function yields the reach.

Proposition 4.3 will be proven in Sect. 4.2, but first we need to refine the upper bound given in item 5. of the properties of $h_{\mathbf{X }}$ given after Definition 4.1 for the case where $\mathbf{X }$ is a submanifold.

### Upper Bounds on the Convexity Defect Function

**Fig. 2:**
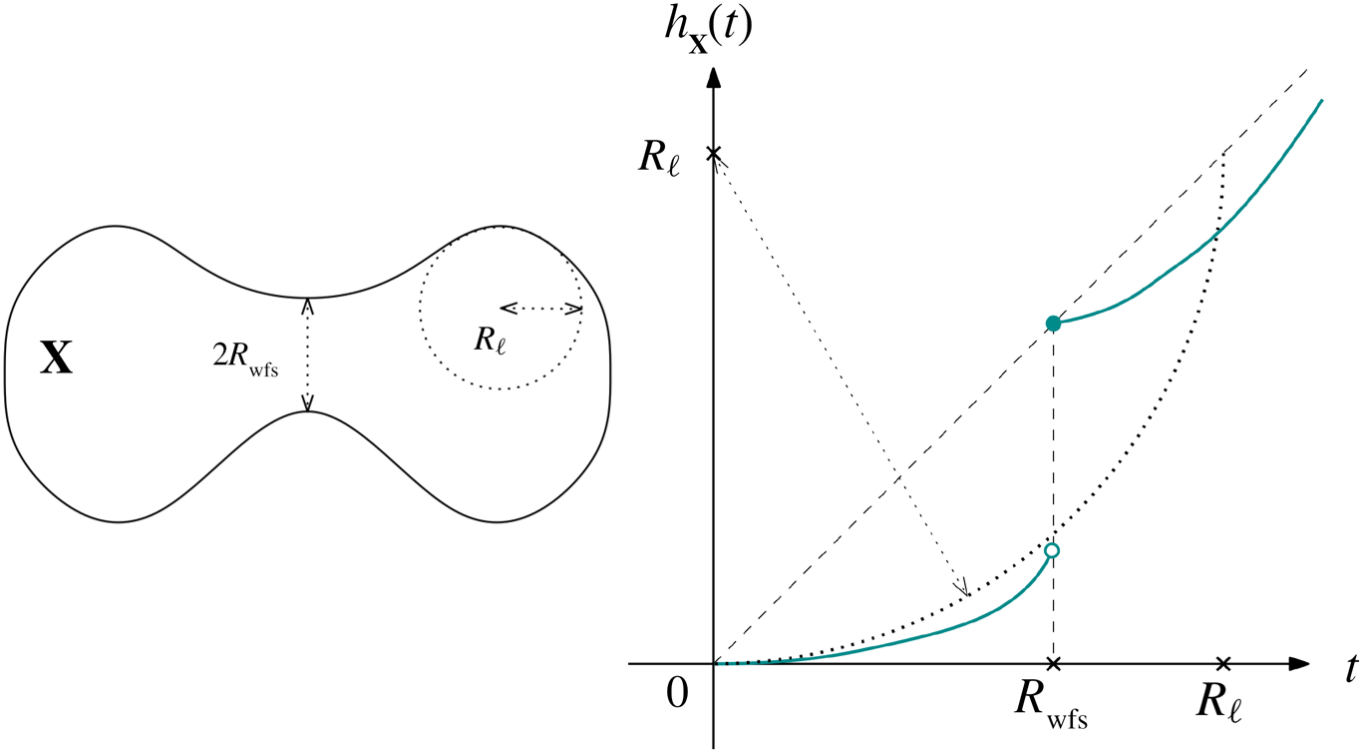
A curve $\mathbf{X }$ (left) and its convexity defect function $h_\mathbf{X } (t)$ (right), which is below the quarter-circle of radius $R_\ell$ for $t < R(\mathbf{X } ) = R_{\mathrm{wfs}}$. Since $R_{\mathrm{wfs}}< R_\ell$, we observe a discontinuity at $t = R_{\mathrm{wfs}}$

The two aspects of the reach relate to the convexity defect function in quite different ways, which naturally leads one to wonder which aspect of the reach is responsible for item 5. of the properties of $h_{\mathbf{X }}$ given after Definition 4.1. In this subsection we improve the upper bound by increasing the radius of the bounding circle from *R* to $R_{\ell }$, though the bound still only holds on the interval [0, *R*) (compare with [6, Lem. 12]). See Fig. 2 for an illustration.

#### Proposition 4.4

If $\mathbf{M } \in {\mathfrak {C}}^k_{R_{\mathrm{min}}, \mathbf{L } }$and $R = R(\mathbf{M } )$ is its reach, then on [0, *R*) the function $h_{\mathbf{M } }(t)$ is bounded above by a quarter-circle of radius $R_{\ell }$ centered on $(0,R_{\ell })$. In other words, $h_{\mathbf{M } }(t) \leqslant R_{\ell } - \sqrt{R_{\ell }^2 - t^2}$.

For submanifolds in the class ${\mathscr {M}}_0^k$ (where $R_{\mathrm{wfs}}\geqslant R_{\ell }$), this result does not have any content. However, for manifolds in ${\mathscr {M}}^k_{\alpha }$, i.e., manifolds for which $R_{\mathrm{wfs}}\leqslant R_{\ell } - \alpha$ for some $\alpha > 0$, the bound is sharper, with the following consequence.

#### Corollary 4.5

If $\mathbf{M } \in {\mathscr {M}}_\alpha ^k$ for some $\alpha > 0$, then $h_{\mathbf{M } }$ is discontinuous at $R(\mathbf{M } )$.

#### Proof

Since $\alpha > 0$, we have $R(\mathbf{M } ) = R_{\mathrm{wfs}}< R_{\ell }$. For $t < R_{\mathrm{wfs}}$ the bound $h_{\mathbf{M } }(t) \leqslant R_{\ell } - \sqrt{R_{\ell }^2 - t^2}$ from Proposition 4.4 holds. On the other hand, for $t = R_{\mathrm{wfs}}$ we have $h_{\mathbf{M } } (t) = t$. Therefore the one-sided limit $\lim _{t\nearrow R_{\mathrm{wfs}}}h_{\mathbf{M } }(t) < h_{\mathbf{M } }(R_{\mathrm{wfs}})$ and the function is discontinuous. $\square$

The proof of Proposition 4.4 will require a few steps. We can focus our attention on the local reach by paying attention to sets of the form $\mathbf{M } ' = \mathbf{M } \cap B(z,r)$, where $z \in \mathbf{R } ^D$, $0< r < R(\mathbf{M } )$, and *B*(*z*, *r*) is a closed ball. Lemma 4.6 will show that subsets of this type have no bottlenecks. We would expect, then, that the reach of such a subset is generated by the local geometry. Lemma 4.9 quantifies this point: the reach of $\mathbf{M } '$ is determined by the behavior of the second fundamental form on $\mathbf{M } '$. The principal point of difficulty here relates to the boundary of the sets $\mathbf{M } '$. The proposition then follows from the fact that $h_{\mathbf{M } }(t)$ can be bounded using the functions $h_{\mathbf{M } '}(t)$ and so the bound is in fact determined by the second fundamental form, i.e., by $R_{\ell }$ in particular.

#### Lemma 4.6

Let $A \subseteq \mathbf{R } ^D$ be a compact set. Let $0< s < R(A)$, $z \in \mathbf{R } ^D$, and $A' = A \cap B(z, s)$, where *B* is a closed ball. If $A'\ne \emptyset$, then $A'$ cannot have any bottlenecks, i.e., there is no pair $p,q \in A'$ with $\Vert p - q \Vert = 2R(A')$ and $(p+q)/2\in {\text {Med}}(A')$.

#### Proof

Suppose for a contradiction that a bottleneck exists. Then it is a chord of length $2R(A')$. Since ${{\,\mathrm{diam}\,}}A' \leqslant 2s$ we obtain that $2R(A') \leqslant 2s < 2 R(A) \leqslant 2R(A')$, the last inequality holding by [5, Lem. 5]. $\square$

We now consider the case where $A= \mathbf{M }$, a submanifold, and consider the intersections $\mathbf{M } '$. Our goal is to find the reach of the intersections, $\mathbf{M } '$, in order to bound $h_{\mathbf{M } '}$ and hence $h_{\mathbf{M } }$. We will use the following characterization of the reach due to Federer [12]:

$$\begin{aligned} \dfrac{1}{R(A)} = \sup _{p,q \in A} \dfrac{2 d(q-p, C_pA)}{\Vert q-p\Vert ^2}, \end{aligned}$$

where $C_pA$ is the tangent cone at *p*, which, as Federer showed, always exists for a set of positive reach. This quotient can be related to the second fundamental form as follows (cf. [1, Lem. 3.3]; and also work of Lytchak [16] for more general results).

#### Lemma 4.7

Let $k \geqslant 3$ and $\mathbf{M } \in {\mathfrak {C}}^k_{R_{\mathrm{min}}, \mathbf{L } }$. Let $\mathbf{M } ' = \mathbf{M } \cap B(z,r)$, where $z \in \mathbf{R } ^D$, $0< r < R(\mathbf{M } )$ and *B* is a closed ball. Then, provided $\mathbf{M } '$ contains more than a single point, for any $p \in \mathbf{M } '$ the norm of the second fundamental form is given by

$$\begin{aligned} \Vert \mathrm{I\,\!I}_p \Vert _{\text {op}} = \limsup _{\begin{array}{c} q \rightarrow p\\ q \in \mathbf{M } ' \end{array}} \dfrac{2 d(q-p, C_p\mathbf{M } ')}{\Vert q-p\Vert ^2}, \end{aligned}$$

where $C_p \mathbf{M } '$ is the cone tangent to $\mathbf{M } '$ at *p*. In particular, $1/R(\mathbf{M } ') \geqslant \sup _{p \in \mathbf{M } '} \Vert \mathrm{I\,\!I}_p\Vert _{\text {op}}$.

#### Proof

We claim that $$\partial \mathbf{M } '$$ is a $${\mathscr {C}}^k$$ submanifold of $$\mathbf{M } $$. Consider the distance function to the central point $$z \in \mathbf{R } ^D$$, say $$f(y) = d(z,y)$$. This function is smooth on $$\mathbf{R } ^D \setminus z$$ and its pull-back $$f| _{\mathbf{M } }$$ is $${\mathscr {C}}^k$$ on $$\mathbf{M } \setminus z$$. For any $$p \in \partial \mathbf{M } '$$, $$ f(p)=r$$. Note that *r* is a critical value of $$f| _{\mathbf{M } }$$ precisely when the distance sphere $$\partial B(z,r)$$ is tangent to $$\mathbf{M } $$ at some $$p\in \mathbf{M } $$.

However, this cannot happen for $r < R(\mathbf{M } )$. This is because *r* is less than the focal radius at *p* and so $\mathbf{M }$ must lie in the exterior of *B*(*z*, *r*). This in turn implies that $\mathbf{M } ' = \{ p\}$ which contradicts the assumption that it is not a singleton. Therefore, *r* is a regular value of the ${\mathscr {C}}^k$ function *f* on $\mathbf{M }$ and the pre-image $\partial \mathbf{M } '$ is an embedded submanifold without boundary, as claimed.

As a consequence, $\mathbf{M } '$ is an embedded submanifold of $\mathbf{M }$ of full dimension with boundary. The tangent cone in $\mathbf{R } ^D$, $C_p \mathbf{M } '$, is given by $T_p \mathbf{M }$ for *p* in the interior of $\mathbf{M } '$ and by a half-space of $T_p \mathbf{M }$ for $p \in \partial \mathbf{M } '$, namely

$$\begin{aligned} C_p \mathbf{M } ' = T_p \mathbf{M } \cap \{u:\langle p-z,u\rangle \leqslant 0\}, \end{aligned}$$

where *z* is the center of the ball containing $\mathbf{M } '$. We now consider some other point $q \in \mathbf{M } '$, $q \ne p$, and show that the projection of *q* to $T_p\mathbf{M }$ lies in $C_p \mathbf{M } '$. Suppose $p \in \partial \mathbf{M } ' \subseteq \partial B$. Consider the affine hyperplane $H^{D-1}$ through *p* perpendicular to the line *pz*. Since $q \in B$, *q* lies on the same side of *H* as *z* and therefore the projection of *q* to $T_p\mathbf{M }$ lies in $C_p \mathbf{M } '$. If $p \notin \partial \mathbf{M } '$ then $T_p \mathbf{M } = C_p \mathbf{M } '$ and so this statement automatically holds.

Let us assume now that *q* is close to *p*, satisfying $\Vert q-p\Vert \leqslant \min {\{R_{\text {min}}, (L_{\perp })^{-1}\}}/4$, so that the projection of *q* to $C_p \mathbf{M } '$ satisfies the conclusion of Lemma 3.2. In particular, if *v* is the projection of *q* onto $T_p\mathbf{M }$, we may write

$$\begin{aligned} q-p = v + \dfrac{\mathrm{I\,\!I}_p(v,v)}{2} + {\mathcal {R}}_3(v), \end{aligned}$$

where the remainder ${\mathcal {R}}_3(v)$ is of order $O(\Vert v\Vert ^3)$. Therefore

$$\begin{aligned} d(q-p, C_p \mathbf{M } ') = \biggl \Vert \dfrac{\mathrm{I\,\!I}_p(v,v)}{2} + {\mathcal {R}}_3(v) \biggr \Vert . \end{aligned}$$

We can then calculate the Federer quotient,

$$\begin{aligned} \dfrac{2 d(q-p, C_p \mathbf{M } ')}{\Vert q-p\Vert ^2}&= \dfrac{\Vert \mathrm{I\,\!I}_p(v,v) + 2 {\mathcal {R}}_3(v) \Vert }{ \Vert v\Vert ^2 + \Vert \mathrm{I\,\!I}_p(v,v)/2+ {\mathcal {R}}_3(v)\Vert ^2}\\&=\dfrac{1}{{\Vert v\Vert ^2}/{\Vert \mathrm{I\,\!I}_p(v,v) + 2 {\mathcal {R}}_3(v) \Vert } + \Vert \mathrm{I\,\!I}_p(v,v) + 2 {\mathcal {R}}_3(v) \Vert /4}. \end{aligned}$$

As $q \rightarrow p$ we see that $v \rightarrow 0$. In order to compute the $\limsup$, we may assume that a sequence of points $q_i$ is chosen so that $\Vert \mathrm{I\,\!I}_p(v_i,v_i)\Vert$ is maximized. Then, since all terms in the denominator go to zero except the ratio ${\Vert v_i\Vert ^2}/{\Vert \mathrm{I\,\!I}_p(v_i,v_i)\Vert }$, we have

$$\begin{aligned} \limsup _{\begin{array}{c} q \rightarrow p\\ q \in \mathbf{M } ' \end{array}} \dfrac{2 d(q-p, C_p\mathbf{M } ')}{\Vert q-p\Vert ^2} = \lim _{i \rightarrow \infty } \dfrac{\Vert \mathrm{I\,\!I}_p(v_i,v_i)\Vert }{\Vert v_i\Vert ^2}. \end{aligned}$$

We would like to claim that

$$\begin{aligned} \lim _{i \rightarrow \infty } \dfrac{\Vert \mathrm{I\,\!I}_p(v_i,v_i)\Vert }{\Vert v_i\Vert ^2}= \Vert \mathrm{I\,\!I}_p \Vert _{\text {op}}, \end{aligned}$$

but recall that *p* may lie on the boundary of $\mathbf{M } '$ and so we must check that a suitable sequence of points $q_i \in \mathbf{M } '$ can be found. Since $C_p \mathbf{M } '$ is a half-space and $\mathrm{I\,\!I}_p$ is a symmetric, bilinear form, there is some unit vector $w \in C_p \mathbf{M } '$ such that $\Vert \mathrm{I\,\!I}_p(w,w)\Vert = \Vert \mathrm{I\,\!I}_p\Vert _{\text {op}}$. Then we can choose a sequence $q_i \in \mathbf{M } '$ so that the projections of the $q_i$ are $t_i v_i$, where the $v_i$ are unit vectors in $C_p \mathbf{M } '$ such that $v_i \rightarrow w$ and the $t_i$ are positive numbers with $t_i \rightarrow 0$. The existence of such a sequence is equivalent to the fact that $w \in C_p\mathbf{M } '$. The final statement then follows from

$$\begin{aligned} {}\Vert \mathrm{I\,\!I}_p \Vert _{\text {op}}= & {} \limsup _{\begin{array}{c} q \rightarrow p\\ q \in \mathbf{M } ' \end{array}} \dfrac{2 d(q-p, C_p\mathbf{M } ')}{\Vert q-p\Vert ^2}\\\leqslant & {} \sup _{p,q \in \mathbf{M } '} \dfrac{2 d(q-p, C_p\mathbf{M } ')}{\Vert q-p\Vert ^2} =\dfrac{1}{R(\mathbf{M } ')}. \end{aligned}$$

$$\square $$

#### Remark 4.8

The regularity assumption of $k \geqslant 3$ in the previous lemma may possibly be improved to $k \geqslant 2$. This stems from the assumption in Lemma 3.2 which in turn derives from the regularity assumption in [3, Lem. 2]. However, this is not needed in the sequel so we do not pursue this further.

#### Lemma 4.9

Let $k \geqslant 3$ and $\mathbf{M } \in {\mathfrak {C}}^k_{R_{\mathrm{min}}, \mathbf{L } }$. Let $\mathbf{M } ' = \mathbf{M } \cap B(z,r)$, where $z \in \mathbf{R } ^D$, $0< r < R(\mathbf{M } )$, and *B* is a closed ball. Then, provided $\mathbf{M } '$ contains more than a single point, we have $1/R(\mathbf{M } ')=\sup _{p \in \mathbf{M } '} \Vert \mathrm{I\,\!I}_p\Vert _{\text {op}}$.

#### Proof

We have already shown in Lemma 4.7 that $1/R(\mathbf{M } ')\geqslant \sup _{p \in \mathbf{M } '} \Vert \mathrm{I\,\!I}_p\Vert _{\text {op}}$. By Lemma 4.6, $\mathbf{M } '$ does not contain any bottlenecks. It follows that the reach is attained in one of two ways and we examine each case.

*Case 1*: The reach of $\mathbf{M } '$ is attained by a pair of points $q,r \in \mathbf{M } '$ but $\Vert q-r \Vert < 2R(\mathbf{M } ')$. In this case we apply [1, Lem. 3.2] to obtain, in $\mathbf{M } '$, an arc of a circle of radius *R* equal to the reach of $\mathbf{M } '$. Note that that lemma is stated for manifolds, but in fact the proof only requires a set of positive reach. Then, for any point *p* on the reach-attaining arc, we obtain that

$$\begin{aligned} \dfrac{1}{R(\mathbf{M } ')} \leqslant \Vert \mathrm{I\,\!I}_p\Vert _{\text {op}} \leqslant \sup _{s \in \mathbf{M } '} \Vert \mathrm{I\,\!I}_s\Vert . \end{aligned}$$

*Case 2*: The reach of $\mathbf{M } '$ is attained at a single point, say *p*, in $\mathbf{M } '$. It follows, using Lemma 4.7, that

$$\begin{aligned} \dfrac{1}{R(\mathbf{M } ')} = \limsup _{\begin{array}{c} q \rightarrow p\\ q \in \mathbf{M } ' \end{array}} \dfrac{2 d(q-p, C_p\mathbf{M } ')}{\Vert q-p\Vert ^2} = \Vert \mathrm{I\,\!I}_p\Vert _{\text {op}} \leqslant \sup _{s \in \mathbf{M } '} \Vert \mathrm{I\,\!I}_s\Vert _{\text {op}}. \end{aligned}$$

Combining the two cases, then, we also have that

$$\begin{aligned} \dfrac{1}{R(\mathbf{M } ')} \leqslant \sup _{s \in \mathbf{M } '} \Vert \mathrm{I\,\!I}_s\Vert _{\text {op}}, \end{aligned}$$

completing the proof. $\square$

#### Proof of Proposition 4.4

Let $\mathbf{M } ' = \mathbf{M } \cap B(z,r)$, where $z \in \mathbf{R } ^D$, $0< r < R(\mathbf{M } )$, and *B* is a closed ball. Recall that on $[0,R(\mathbf{M } '))$ we have

$$\begin{aligned} h_{\mathbf{M } '}(t) \leqslant R(\mathbf{M } ') - \sqrt{R(\mathbf{M } ')^2 - t^2}\,. \end{aligned}$$

By Lemma 4.9, if $\mathbf{M } '$ is not a single point we have

$$\begin{aligned} \dfrac{1}{R_{\ell }} = \sup _{s \in \mathbf{M } } \Vert \mathrm{I\,\!I}_s\Vert _{\text {op}} \geqslant \sup _{s \in \mathbf{M } '} \Vert \mathrm{I\,\!I}_s\Vert _{\text {op}} = \dfrac{1}{R(\mathbf{M } ')}, \end{aligned}$$

and this entails the bound $h_{\mathbf{M } '}(t) \leqslant R_{\ell } - \sqrt{R_{\ell }^2 - t^2}$ on $[0,R(\mathbf{M } '))$. If $\mathbf{M } '$ is a point then $h_{\mathbf{M } '}(t) = 0$ for all *t* and so the same bound holds.

Recalling that $R(\mathbf{M } ') \geqslant R(\mathbf{M } )$ for every $\mathbf{M } '$ with ${{\,\mathrm{Rad}\,}}\mathbf{M } '<R(\mathbf{M } )$, we have, for $0< t \leqslant r < R(\mathbf{M } )$,

$$\begin{aligned} \sup _{\mathbf{M } ' = \mathbf{M } \cap B(z,r)}\!\!h_{\mathbf{M } '}(t) \leqslant R_{\ell } - \sqrt{R_{\ell }^2 - t^2}. \end{aligned}$$

Now for every $\sigma \subset \mathbf{M }$ with ${{\,\mathrm{Rad}\,}}\sigma \leqslant t \leqslant r$, there is some $\mathbf{M } ' = \mathbf{M } \cap B(z,r)$ with $\sigma \subset \mathbf{M } '$ and it follows that

$$\begin{aligned} h_{\mathbf{M } }(t) \leqslant \sup _{\mathbf{M } ' = \mathbf{M } \cap B(z,r)} \!\!h_{\mathbf{M } '}(t). \end{aligned}$$

Setting $t=r$ and combining the two inequalities, we have, for $0< t < R(\mathbf{M } )$,

$$\begin{aligned} h_{\mathbf{M } }(t) \leqslant R_{\ell } - \sqrt{R_{\ell }^2 - t^2}\,. \end{aligned}$$

$$\square $$

### The Convexity Defect Function Near Zero

We have seen in the previous section how, for $\mathbf{M } \subseteq \mathbf{R } ^D$ a compact submanifold, the function $h_{\mathbf{M } }$ on [0, *R*) obeys an upper bound determined by $R_{\ell }$. We now study $h_{\mathbf{M } }$ in greater detail in a neighborhood of zero to obtain a Taylor polynomial, identifying $R_{\ell }$ as the reciprocal of the ‘pointwise second derivative’, $1/h''_{\mathbf{M } }(0)$. More formally, we prove Proposition 4.3, which states that, for any sufficiently small *t*,

$$\begin{aligned} \biggl |h_{\mathbf{M } }(t) - \dfrac{t^2}{2 R_\ell } \biggr | \leqslant Ct^{k \wedge 4}. \end{aligned}$$

Once more, we approach $h_{\mathbf{M } }$ by considering sets $\mathbf{M } '$, which are the intersection of $\mathbf{M }$ with small closed balls.

We introduce a new function $h_{\mathbf{M } '}^{{\text {loc}}}(p,r_1,r_2 ; t)$, which contains information on the convexity of $\mathbf{M } '$. Lemma 4.10 shows how $h_{\mathbf{M } }$ can be determined from all the $h_{\mathbf{M } '}^{{\text {loc}}}(p,r_1,r_2 ; t)$. Recall from Lemma 3.2 that such sets $\mathbf{M } '$ can be written as the graphs of functions over $T_p \mathbf{M }$ and that these functions have Taylor expansions. Lemma 4.12 will set a lower bound on $h^{\mathrm{loc}}$ for the degree 3 approximation to $\mathbf{M }$ around *p*, which Lemma 4.14 translates to a lower bound on $h_{\mathbf{M } '}^{{\text {loc}}}(p,r_1,r_2 ; t)$ itself. Varying $\mathbf{M } '$ we obtain a lower bound on $h_{\mathbf{M } }(t)$ for small *t*, which we combine with the upper bound from Proposition 4.4 to prove the result.

#### Lemma 4.10

Let *B* denote a closed ball. Then, for any compact set $\mathbf{X} \subset {\mathbf{R }}^D$ and any $r_1 ,r_2,t > 0$ satisfying $r_1 \geqslant 2t$ and $r_2 \geqslant t+r_1$, we have

$$\begin{aligned} h_{\mathbf{X } }(t) = \sup _{p \in \mathbf{X } } h_{\mathbf{X } }^{{\text {loc}}}(p,r_1,r_2 ; t) \end{aligned}$$

where

$$\begin{aligned} h_{\mathbf{X } }^{{\text {loc}}}(p,r_1,r_2 ; t) \,=\, {{\,\mathrm{H}\,}}\left( \bigcup _{\begin{array}{c} \sigma \subseteq \mathbf{X } \cap B(p,r_1)\\ {{\,\mathrm{Rad}\,}}\sigma \leqslant t \end{array}}\!\!\! {{\,\mathrm{Hull}\,}}\sigma \;\Bigg |\;\mathbf{X } \cap B(p,r_2) \right) \!. \end{aligned}$$

#### Proof

We begin by showing $h_\mathbf{X }(t) \geqslant \sup _{p \in \mathbf{X } } h_\mathbf{X }^{{\text {loc}}}(p,r_1,r_2 ; t)$. We have immediately, for any $p \in \mathbf{X }$ and any $r_1,t > 0$,

$$\begin{aligned} h_\mathbf{X }(t) \,=\, {\text {H}}\left( \bigcup _{\begin{array}{c} \sigma \subseteq \mathbf{X }\\ {{\,\mathrm{Rad}\,}}\sigma \leqslant t \end{array}}\!\! {{\,\mathrm{Hull}\,}}\sigma \;\Bigg |\; \mathbf{X } \right) \geqslant \, {\text {H}}\left( \bigcup _{\begin{array}{c} \sigma \subseteq \mathbf{X } \cap B(p,r_1)\\ {{\,\mathrm{Rad}\,}}\sigma \leqslant t \end{array}} \!\!\!{{\,\mathrm{Hull}\,}}\sigma \;\Bigg |\; \mathbf{X } \right) \!, \end{aligned}$$

and so all that is necessary is to check that

$$\begin{aligned}&{\text {H}}\left( \bigcup _{\begin{array}{c} \sigma \subseteq \mathbf{X } \cap B(p,r_1)\\ {{\,\mathrm{Rad}\,}}\sigma \leqslant t \end{array}} \!\!\!{{\,\mathrm{Hull}\,}}\sigma \;\Bigg |\; \mathbf{X } \right) \\&\quad =\, {\text {H}}\left( \bigcup _{\begin{array}{c} \sigma \subseteq \mathbf{X } \cap B(p,r_1)\\ {{\,\mathrm{Rad}\,}}\sigma \leqslant t \end{array}} \!\!\!{{\,\mathrm{Hull}\,}}\sigma \;\Bigg |\; {\mathbf{X }\cap B(p,r_2)} \right) =\,h_\mathbf{X }^{{\text {loc}}}(p,r_1,r_2 ; t). \end{aligned}$$

Let the asymmetric distance

$$\begin{aligned} {{\,\mathrm{H}\,}}\left( \bigcup _{\begin{array}{c} \sigma \subseteq \mathbf{X } \cap B(p,r_1)\\ {{\,\mathrm{Rad}\,}}\sigma \leqslant t \end{array}} \!\!\!{{\,\mathrm{Hull}\,}}\sigma \; \Bigg |\; \mathbf{X } \right) \end{aligned}$$

be realized by the data $\sigma \subseteq \mathbf{X } \cap B(p,r_1)$, $y \in {{\,\mathrm{Hull}\,}}\sigma$, $p' \in \mathbf{X }$. We have $d(p',y) \leqslant t$ and $d(y,p) \leqslant r_1$, so that $d(p',p) \leqslant r_1+t \leqslant r_2$.

Now we check that $h_\mathbf{X }(t) \leqslant \sup _{p \in \mathbf{X } } h_\mathbf{X }^{{\text {loc}}}(p,r_1,r_2 ; t)$. If $\sigma \subset \mathbf{X }$, we have $\sigma \subset B(p, 2 {{\,\mathrm{Rad}\,}}\sigma )$ for any $p\in \sigma$. By requiring ${{\,\mathrm{Rad}\,}}\sigma \leqslant t$, we obtain ${{\,\mathrm{H}\,}}({{\,\mathrm{Hull}\,}}\sigma |\mathbf{X }) \leqslant h_\mathbf{X }^{{\text {loc}}}(p,r_1,r_2 ; t)$ for any $p \in \sigma$ provided $r_1 \geqslant 2t$. $\square$

For a bilinear map $S:\mathbf{R }^d \times \mathbf{R }^d \rightarrow \mathbf{R }^{D-d}$ and a trilinear map $T:\mathbf{R }^d \times \mathbf{R }^d \times \mathbf{R }^d \rightarrow \mathbf{R }^{D-d}$, we denote

$$\begin{aligned} M(S,T) =\bigl \{\bigl (v,S(v^{\otimes 2})+T(v^{\otimes 3})\bigr ):v\in \mathbf{R }^d\bigr \} \subseteq \mathbf{R }^D, \end{aligned}$$

which is a ${\mathscr {C}}^\infty$ submanifold of $\mathbf{R }^D$ of dimension *d*.

By setting *S* and *T* to be the coefficients of $\Phi ^3_p$, the approximation of degree 3 to a manifold $\mathbf{M }$ around $p \in \mathbf{M }$ (see Definition 3.3), we can easily see that, near *p*, *M*(*S*, *T*) is Hausdorff close to $\mathbf{M }$. This assumes that $p=0$ and that $T_p \mathbf{M }$ is the subspace spanned by the first *d* coordinates. This assumption, which is used in the statement of the lemma below, is for convenience only. For each $p \in \mathbf{M }$ there is an isometry of $\mathbf{R } ^D$ which causes it to be satisfied.

#### Lemma 4.11

Let $\mathbf{M } \in {\mathfrak {C}}^k_{R_{\mathrm{min}}, \mathbf{L } }$. Suppose that $p=0 \in \mathbf{M }$ and $T_p \mathbf{M } = \mathbf{R } ^d \subseteq \mathbf{R } ^D$.

- If $k \geqslant 4$, we have, for $s \leqslant s_{1}$ with $s_1$ depending only on $R_{\mathrm{min}}, \mathbf{L } , k ,d$,
  $$\begin{aligned} {{\,\mathrm{H}\,}}( \mathbf{M } \cap B(0,s), M(S,T)\cap B(0,s)) \leqslant C s^4, \end{aligned}$$
  where *S* and *T* are obtained from the degree 3 approximation $\Phi _0^3$ given in Definition 3.3 by $S = \mathrm{d}_0^2 \Phi _0^3 / 2=\mathrm{I\,\!I}_0$, $T = \mathrm{d}_0^3 \Phi _0^3 / 6$, and the constant $C = C_{R_{\mathrm{min}}, \mathbf{L } , k ,d}$.
- When $k=3$ we can use the degree 2 approximation $\Phi _0^2$ and pick $T \equiv 0$, to obtain
  $$\begin{aligned} {{\,\mathrm{H}\,}}( \mathbf{M } \cap B(0,s), M(S,0)\cap B(0,s)) \leqslant C' s^3. \end{aligned}$$

#### Proof

Let us initially take $s_1 = \min {\lbrace R_{\mathrm{min}}, L_{\perp }^{-1}\rbrace }/4$. Then for any point $q \in \mathbf{M } \cap B(0,s)$, if $v = {{\,\mathrm{pr}\,}}_{T_0 \mathbf{M } }(q)$ then

$$\begin{aligned} q=\Phi _0(v) = v + S(v^{\otimes 2}) + T(v^{\otimes 3})+{\mathcal {R}}(v), \end{aligned}$$

where $\Phi _0$ is the expansion given in Lemma 3.2 and $\Vert {\mathcal {R}}(v)\Vert \leqslant {L'_4}\Vert v\Vert ^4\!/{24}$, unless $k=3$. In case $k=3$, if we wish to control the remainder we can only use the degree 2 polynomial approximation $\Phi _0^2$.

It is therefore clear that, for the point $q = \Phi _0(v) \in \mathbf{M } \cap B(0,s)$, there is a corresponding point $\Phi _0^3(v) \in M(S,T)$ within the required distance and, conversely, for any point $\Phi _0^3(v) \in M(S,T) \cap B(0,s)$, there is a corresponding point $\Phi _0(v) \in \mathbf{M }$. The constant *C* may be chosen to be $C = {L'_4}/{24}$. However, the corresponding point is not guaranteed to lie in the ball *B*(0, *s*). In the next paragraph we establish that there is a vector $v'$ very close to *v*, such that $\Phi _0^3(v')$ or $\Phi _0(v')$, as appropriate, will be sufficiently close.

Let us continue to assume $$k \geqslant 4$$, since the case $$k=3$$ is essentially identical. We first consider the possibility that $$\Vert \Phi _0^3(v) \Vert \leqslant s$$ but $$\Vert \Phi _0(v) \Vert > s$$ . It is clear that, for sufficiently small *s*, $$\Vert \Phi _0(v) \Vert ^2 \leqslant s^2 + C_0 s^6$$, where $$C_0 $$ depends on $$R_{\mathrm{min}}$$, $$L_{\perp }$$, $$L_3$$, and $$L_4$$. It follows that $$\Vert \Phi _0(v) \Vert \leqslant s + C_1 s^5$$. Consider now a vector $$v' = (1-\lambda ) v$$, with $$\lambda \approx 0$$, chosen so that $$\Vert \Phi _0(v') \Vert =s$$. For small enough *s* we have $$\lambda \leqslant C_2 s^4$$. It follows immediately that $$\Phi _0(v')$$ lies within $$C_3 s^4$$ of $$\Phi _0(v)$$, and hence within $$C s^4$$ of $$\Phi _0^3(v)$$. The case where $$\Vert \Phi _0(v) \Vert \leqslant s$$ but $$\Vert \Phi _0^3(v) \Vert > s$$ is dealt with similarly. $$\square $$

The utility of *M*(*S*, *T*) is that, since it is algebraic, we can compute explicit bounds for $h_{\mathbf{X } }^{{\text {loc}}}$, where $\mathbf{X } = M(S,T)$.

#### Lemma 4.12

Let $r_1 \leqslant r_2 \leqslant {13^{1/4}}\Vert T\Vert _{{{\,\mathrm{op}\,}}}^{-1/2}\!/2$, and let $\mathbf{X } =M(S,T)$. Then for any $t \leqslant \min {\{\Vert S\Vert _{{{\,\mathrm{op}\,}}}^{-1}/2,{2}r_1/\!{\sqrt{13}}\}}$ we have

$$\begin{aligned} h_{\mathbf{X } }^{{\text {loc}}}(0,r_1,r_2 ; t) \geqslant \biggl (t - \dfrac{t^5 \Vert T\Vert _{{{\,\mathrm{op}\,}}}^2}{2}\biggr )^{\!2}\Vert S\Vert _{{{\,\mathrm{op}\,}}}\geqslant t^2 \Vert S\Vert _{{{\,\mathrm{op}\,}}}- t^6 \Vert S\Vert _{{{\,\mathrm{op}\,}}}\Vert T\Vert _{{{\,\mathrm{op}\,}}}^2. \end{aligned}$$

#### Proof

Let *v* be a unit norm vector in $\mathbf{R }^d$ such that $\Vert S(v^{\otimes 2})\Vert = \Vert S\Vert _{{{\,\mathrm{op}\,}}}$. Let $z \leqslant \min {\{\Vert S\Vert _{{{\,\mathrm{op}\,}}}^{-1}/2,{2}r_1/\!{\sqrt{13}}\}}$. Note that the upper bound on $r_1$ gives a third upper bound for *z*, namely $z \leqslant 13^{-1/4} \Vert T\Vert _{{{\,\mathrm{op}\,}}}^{-1/2} \leqslant \Vert T\Vert _{{{\,\mathrm{op}\,}}}^{-1/2}$. We set

$$\begin{aligned}&p_1 = \bigl (zv,S((zv)^{\otimes 2})+T((zv)^{\otimes 3})\bigr )\quad \text {and} \\&p_2 = \bigl (-zv,S((-zv)^{\otimes 2})+T((-zv)^{\otimes 3})\bigr ) \end{aligned}$$

and denote the two-point set containing them by $\sigma = \{p_1,p_2\}$. In order to use $\sigma$ to bound $h_{\mathbf{X } }^{{\text {loc}}}$ we must (1) check $\sigma \subseteq \mathbf{X } \cap B(0,r_1)$, (2) find the radius of $\sigma$, and (3) determine ${{\,\mathrm{H}\,}}({{\,\mathrm{Hull}\,}}\sigma \,|\, \mathbf{X } \cap B(0,r_2))$.

Firstly, since $\sigma \subseteq M(S,T)$, it is enough to show that $\Vert p_1\Vert ^2, \Vert p_2\Vert ^2 \leqslant r_1^2$. Using all three bounds on *z*, we can check

$$\begin{aligned} \Vert p_1\Vert ^2, \Vert p_2\Vert ^2&\leqslant z^2+z^4 \Vert S\Vert _{{{\,\mathrm{op}\,}}}^2+ 2z^5\Vert S\Vert _{{{\,\mathrm{op}\,}}}\Vert T\Vert _{{{\,\mathrm{op}\,}}}+z^6\Vert T\Vert _{{{\,\mathrm{op}\,}}}^2&\\&\leqslant 2z^2+2z^3\Vert S\Vert _{{{\,\mathrm{op}\,}}}+ z^4 \Vert S\Vert _{{{\,\mathrm{op}\,}}}^2&\text {by }z \Vert T\Vert _{{{\,\mathrm{op}\,}}}^{1/2}<1 \\&\leqslant \dfrac{13}{4} z^2&\text {by }z \Vert S\Vert _{{{\,\mathrm{op}\,}}}\leqslant {1}/{2}\\&\leqslant r_1^2&\text {by }z \leqslant {2}r_1/\!{\sqrt{13}}. \end{aligned}$$

Secondly, we obtain the radius as

$$\begin{aligned} {{\,\mathrm{Rad}\,}}\sigma&=\dfrac{\sqrt{(2z)^2+(2z^3\Vert T(v^{\otimes 3})\Vert )^2}}{2}=z\sqrt{1+z^4\Vert T(v^{\otimes 3})\Vert ^2}\\&\leqslant z\biggl (1+\dfrac{z^4\Vert T\Vert _{{{\,\mathrm{op}\,}}}^2}{2}\biggr )\qquad \qquad \text {since }\ \sqrt{1+x} \leqslant 1+ \dfrac{x}{2}\ \text { for }x \geqslant 0\\&= z+\dfrac{z^5\Vert T\Vert _{{{\,\mathrm{op}\,}}}^2}{2}.&\end{aligned}$$

Thirdly, we place a lower bound on ${{\,\mathrm{H}\,}}({{\,\mathrm{Hull}\,}}\sigma \,|\,\mathbf{X } \cap B(0,r_2) )$. Let $q=(p_1+p_2)/2 \in {{\,\mathrm{Hull}\,}}\sigma$. For any $p = (w,S(w^{\otimes 2})+T(w^{\otimes 3})) \in \mathbf{X }$ satisfying $\Vert w\Vert \leqslant r_2$, we have

$$\begin{aligned} d(q,p)^2&= \Vert w\Vert ^2 + \Vert S(w^{\otimes 2})+T(w^{\otimes 3}) - z^2 S(v^{\otimes 2})\Vert ^2 \\&\geqslant z^4 \Vert S\Vert _{{{\,\mathrm{op}\,}}}^2+\Vert w\Vert ^2\bigl (1 - 2z^2\Vert S\Vert _{{{\,\mathrm{op}\,}}}^2 - 2z^2 r_2\Vert S\Vert _{{{\,\mathrm{op}\,}}}\Vert T\Vert _{{{\,\mathrm{op}\,}}}\bigr ). \end{aligned}$$

Since $z\Vert S\Vert _{{{\,\mathrm{op}\,}}}\leqslant 1/{2}$ we have $2z^2\Vert S\Vert _{{{\,\mathrm{op}\,}}}^2 \leqslant 1/2$. The same condition also allows us to see that $2z^2r_2\Vert S\Vert _{{{\,\mathrm{op}\,}}}\Vert T\Vert _{{{\,\mathrm{op}\,}}}\leqslant zr_2\Vert T\Vert _{{{\,\mathrm{op}\,}}}\leqslant 1/2$. It follows that

$$\begin{aligned} d(q,p)^2 \geqslant z^4 \Vert S\Vert ^2_{{{\,\mathrm{op}\,}}} = d(q,0)^2 \end{aligned}$$

from which we obtain the bound ${{\,\mathrm{H}\,}}({{\,\mathrm{Hull}\,}}\sigma \,|\, \mathbf{X } \cap B(0,r_2)) \geqslant z^2 \Vert S\Vert _{{{\,\mathrm{op}\,}}}$.

These three calculations yield $h_\mathbf{X } ^{{\text {loc}}}(0,r_1,r_2 ; z+z^5\Vert T\Vert _{{{\,\mathrm{op}\,}}}^2/2) \geqslant z^2 \Vert S\Vert _{{{\,\mathrm{op}\,}}}$. Now we may reparametrize the argument by setting $t=z+z^5 \Vert T\Vert _{{{\,\mathrm{op}\,}}}^2/2$. Obviously $t \geqslant z$ so we can invert to obtain $z = t -z^5 \Vert T\Vert _{{{\,\mathrm{op}\,}}}^2 /2\geqslant t -t^5 \Vert T\Vert _{{{\,\mathrm{op}\,}}}^2/2$, and so $h_\mathbf{X } ^{{\text {loc}}}(0,r_1,r_2;t) \geqslant (t -t^5 \Vert T\Vert _{{{\,\mathrm{op}\,}}}^2/2)^2 \Vert S\Vert _{{{\,\mathrm{op}\,}}}\geqslant (t^2 - t^6 \Vert T\Vert _{{{\,\mathrm{op}\,}}}^2) \Vert S\Vert _{{{\,\mathrm{op}\,}}}$. If the bounds given in the statement hold for *t*, then they will also hold for the smaller value *z* and so the result is proven.

$$\square $$

We are now in a position to convert this bound for an algebraic approximation to $\mathbf{M }$ into one for the small patch of $\mathbf{M }$ itself. We need a stability result first.

#### Lemma 4.13

Let $\mathbf{X } , \mathbf{Y }$ be two subsets of $\mathbf{R}^D$ and let $r_1, r_2 ,t > 0$ be such that $r_1 \leqslant r_2$. Then, if $p \in \mathbf{X } \cap \mathbf{Y }$ and ${{\,\mathrm{H}\,}}(\mathbf{X } \cap B(p,r_2),\mathbf{Y } \cap B(p,r_2)) \leqslant \varepsilon$, we have

$$\begin{aligned} h_{\mathbf{X } }^{{\text {loc}}}(p,r_1,r_2 ; t) \leqslant h^{{\text {loc}}}_{\mathbf{Y } }(p,r_1 + \varepsilon , r_2 ; t + \varepsilon ) + 2\varepsilon . \end{aligned}$$

#### Proof

This is a straightforward adaptation of the proof of [6, Lem. 5]. Indeed, let $\sigma \subset \mathbf{X } \cap B(p,r_1)$ be such that ${{\,\mathrm{Rad}\,}}\sigma \leqslant t$. Let $\xi = \mathbf{Y } \cap B(p,r_2) \cap \sigma ^\varepsilon$. Since ${{\,\mathrm{H}\,}}(\mathbf{X } \cap B(p,r_2), \mathbf{Y } \cap B(p,r_2)) \leqslant \varepsilon$, $\xi$ is not empty and satisfies ${{\,\mathrm{H}\,}}(\xi , \sigma ) \leqslant \varepsilon$. Thus $\xi \subset \mathbf{Y } \cap B(p,r_1+\varepsilon )$, and furthermore, by [6, Lem. 16], we have ${{\,\mathrm{Rad}\,}}\xi \leqslant t + \varepsilon$. We conclude using that

$$\begin{aligned} {{\,\mathrm{Hull}\,}}\sigma \subset {{\,\mathrm{Hull}\,}}\xi ^\varepsilon&= ({{\,\mathrm{Hull}\,}}\xi )^\varepsilon \subset (\mathbf{Y } \cap B(p,r_2))^{h^{{\text {loc}}}_{\mathbf{Y } } (p,r_1 +\varepsilon , r_2 ; t + \varepsilon ) +\varepsilon } \\&\subset (\mathbf{X } \cap B(p,r_2))^{h^{{\text {loc}}}_{\mathbf{Y } } (p,r_1 +\varepsilon , r_2 ; t + \varepsilon ) +2\varepsilon }. \end{aligned}$$

$$\square $$

#### Lemma 4.14

Let $k \geqslant 4$. There exists $s_2 > 0$ depending only on $R_{\mathrm{min}}, \mathbf{L } , k, d$ such that for any $r_2 \leqslant s_2$ and for any $r_1, t \geqslant 0$ such that both $r_1 \leqslant r_2$ and

$$\begin{aligned} C_0 r_2^4 \leqslant t \leqslant \dfrac{2}{\sqrt{13}} r_1 \end{aligned}$$

for some constant $C_0$ depending on $R_{\mathrm{min}},\mathbf{L } , k ,d$, we have, for all $\mathbf{M } \in {\mathfrak {C}}^k_{R_{\mathrm{min}}, \mathbf{L } }$ and all $p \in \mathbf{M }$,

$$\begin{aligned} h_{\mathbf{M } }^{{\text {loc}}}(p,r_1,r_2 ; t) \geqslant \dfrac{t^2 \Vert \mathrm{I\,\!I}_p\Vert _{{{\,\mathrm{op}\,}}}}{2} - C r_2^4, \end{aligned}$$

where *C* is a constant depending on $R_{\mathrm{min}},\mathbf{L } , k ,d$. In case $k=3$, we have, for all $\mathbf{M } \in {\mathfrak {C}}^k_{R_{\mathrm{min}}, \mathbf{L } }$ and all $p \in \mathbf{M }$,

$$\begin{aligned} h_{\mathbf{M } }^{{\text {loc}}}(p,r_1,r_2 ; t) \geqslant \dfrac{t^2 \Vert \mathrm{I\,\!I}_p\Vert _{{{\,\mathrm{op}\,}}}}{2} - C' r_2^3, \end{aligned}$$

where $C'$ is a constant depending on $R_{\mathrm{min}},\mathbf{L } , d$.

#### Proof

By applying an isometry of $\mathbf{R } ^D$, we may assume that $p=0$ and that $T_p \mathbf{M } = \mathbf{R } ^d \subseteq \mathbf{R } ^D$. The result will then follow from Lemmata 4.11 and 4.12 in addition to the Hausdorff stability property for $h^{{\text {loc}}}$ (Lemma 4.13). Take $r_2 > 0$ smaller than $s_1$ (from Lemma 4.11), and than ${13^{1/4}}/({2{L'_3}^{1/2}})$ (from Lemma 4.12). In the case $k \geqslant 4$, where $\Phi _p$ is the expansion described in Lemma 3.2, $S=\mathrm{d}_0^2\Phi _p/2=\mathrm{I\,\!I}_p$, $T=\mathrm{d}_0^3\Phi _p/6$, and $C_0$ is the constant from the statement of Lemma 4.11, we have

$$\begin{aligned} h_{\mathbf{M } }^{{\text {loc}}}(0,r_1,r_2; t)&\geqslant h_{M(S,T)}^{{\text {loc}}}(0,r_1 - C_0 s^{4},r_2 ; t - C_0 r_2^4) - 2C_0 r_2^4 \\&\geqslant (t - C_0 r_2^4)^2 \Vert S\Vert _{{{\,\mathrm{op}\,}}}- (t - C_0 r_2^4)^6 \Vert S\Vert _{{{\,\mathrm{op}\,}}}\Vert T\Vert _{{{\,\mathrm{op}\,}}}^2 - 2C_0 r_2^4\\&\geqslant \dfrac{ t^2\Vert \mathrm{I\,\!I}_p\Vert _{{{\,\mathrm{op}\,}}} }{2} - C r_2^4, \end{aligned}$$

where *C* depends only on $R_{\min }, \mathbf{L } , d, k$. The first inequality only holds if $C_0 r_2^4 \leqslant t$. In the case $k=3$ the result is obtained similarly. $\square$

We conclude with the proof of Proposition 4.3.

#### Proof of Proposition 4.3

By taking

$$\begin{aligned} t \leqslant \dfrac{s_2}{4} \wedge (4^4 C_0)^{-1/3} \end{aligned}$$

(from Lemma 4.14), and setting $r_1=2t$ and $r_2 = 3t$, we have

$$\begin{aligned} C_0 r_2^4 \leqslant t \leqslant \dfrac{2}{\sqrt{13}} r_1 \quad \ \text {and} \quad \ t+r_1 \leqslant r_2, \end{aligned}$$

so that the hypotheses of both Lemmata 4.10 and 4.14 hold. It is now immediate that if $k \geqslant 4$,

$$\begin{aligned} h_{\mathbf{M } }(t) = \sup _{p \in \mathbf{M } } \,{h_{\mathbf{M } }^{{\text {loc}}}(p,r_1,r_2;t) }\geqslant \sup _{p \in \mathbf{M } } {\biggl (\dfrac{t^2 \Vert \mathrm{I\,\!I}_p\Vert _{{{\,\mathrm{op}\,}}}}{2} - C r_2^4\biggr )}=\dfrac{t^2}{2R_{\ell }} - 3^4 C t^4, \end{aligned}$$

where *C* is a constant depending on $R_{\mathrm{min}},\mathbf{L } ,d,k$, while if $k=3$,

$$\begin{aligned} h_{\mathbf{M } }(t) \geqslant \dfrac{t^2}{2R_{\ell }} - C' t^3, \end{aligned}$$

where $C'$ is a constant depending on $R_{\mathrm{min}},\mathbf{L }$. On the other hand, Proposition 4.4 provides an upper bound which will hold for all $t < R_{\mathrm{min}}$:

$$\begin{aligned} {}h_\mathbf{M } (t)\leqslant R_\ell - \sqrt{R_\ell ^2 - t^2} \leqslant \dfrac{t^2}{2R_\ell } + \dfrac{t^4}{2R_\ell ^3}\leqslant \dfrac{t^2}{2R_\ell } + \dfrac{t^4}{2R_{\mathrm{min}}^3}. \end{aligned}$$

$$\square $$

## Approximating the Reach

Recall item 3. of the properties of $h_{\mathbf{X }}$ given after Definition 4.1 which guarantees that the convexity defect function is stable with respect to perturbations of the manifold which are small in the Hausdorff distance. This allows one to approximate the reach of a submanifold $\mathbf{M } \subseteq \mathbf{R } ^D$ from a nearby subset ${\widetilde{\mathbf{M } }}$.

Given a submanifold $\mathbf{M }$ and another subset ${\widetilde{\mathbf{M } }}$ (not necessarily a manifold) so that H$(\mathbf{M } , {\widetilde{\mathbf{M } }}) < \varepsilon$, we can calculate the convexity defect function $h_{{\widetilde{\mathbf{M } }}}$. This can then be used to approximate $R_{\ell } = ( h_{\mathbf{M } }''(0))^{-1}$ and $R_{\mathrm{wfs}}= \inf {\lbrace t: h_{\mathbf{M } }(t)=t, \,t>0\rbrace }$. We can approximate the local reach via

$$\begin{aligned} h_{\mathbf{M } }''(0) \approx 2\dfrac{h_{{\widetilde{\mathbf{M } }}}(\Delta )}{\Delta ^2} \end{aligned}$$

for some choice of step size $\Delta$. Proposition 4.3 gives the following bound on the error.

### Proposition 5.1

Let $\mathbf{M } \in {\mathfrak {C}}^k_{R_{\mathrm{min}}, \mathbf{L } }$. Let $0< \varepsilon< \Delta < 1$ be such that $\varepsilon + \Delta$ is small enough to satisfy the hypotheses constraining the variable *t* in Proposition 4.3. Let ${\widetilde{\mathbf{M } }}\subseteq \mathbf{R } ^D$ be such that H$(\mathbf{M } , {\widetilde{\mathbf{M } }}) < \varepsilon$. Then

- if $k \geqslant 4$, $\bigl | h_{\mathbf{M } }''(0) - 2{h_{{\widetilde{\mathbf{M } }}}(\Delta )}/{\Delta ^2}\bigr | \leqslant A \varepsilon \Delta ^{-2}+ B \Delta ^2$ and, in particular, if $\Delta = \varepsilon ^{1/4}$,
  $$\begin{aligned} \biggl | h_{\mathbf{M } }''(0) - 2\dfrac{h_{{\widetilde{\mathbf{M } }}}(\Delta )}{\Delta ^2}\biggr | \leqslant (A+B)\varepsilon ^{1/2}, \end{aligned}$$
- if $k=3$, $\bigl | h_{\mathbf{M } }''(0) - 2{h_{{\widetilde{\mathbf{M } }}}(\Delta )}/{\Delta ^2}\bigr | \leqslant A \varepsilon \Delta ^{-2}+ B \Delta$ and, in particular, if $\Delta = \varepsilon ^{1/3}$,
  $$\begin{aligned} \biggl | h_{\mathbf{M } }''(0) - 2\dfrac{h_{{\widetilde{\mathbf{M } }}}(\Delta )}{\Delta ^2}\biggr | \leqslant (A+B) \varepsilon ^{1/3}, \end{aligned}$$

where the constants *A* and *B* depend only on $R_{\mathrm{min}},\mathbf{L }$.

### Proof

Set $\kappa = h_{\mathbf{M } }''(0)$ and ${\tilde{\kappa }} = 2{h_{{\widetilde{\mathbf{M } }}}(\Delta )}/{\Delta ^2}$. Comparing $\mathbf{M }$ to ${\widetilde{\mathbf{M } }}$, we obtain from stability that

$$\begin{aligned} 2\dfrac{h_{\mathbf{M } }(\Delta -\varepsilon ) - 2 \varepsilon }{\Delta ^2}\leqslant {\tilde{\kappa }}\leqslant 2\dfrac{h_{\mathbf{M } }(\Delta +\varepsilon ) + 2 \varepsilon }{\Delta ^2}. \end{aligned}$$

In the case $k \geqslant 4$, Proposition 4.3 states that $| h_{\mathbf{M } }(t) - {\kappa }t^2/2| \leqslant Ct^4$, for some constant *C* depending only on $R_{\mathrm{min}},\mathbf{L }$. It follows that

$$\begin{aligned} \dfrac{\kappa (\Delta - \varepsilon )^2 - 2C(\Delta - \varepsilon )^4 - 4\varepsilon }{\Delta ^2}\leqslant {\tilde{\kappa }}\leqslant \dfrac{\kappa (\Delta + \varepsilon )^2 + 2C(\Delta + \varepsilon )^4 + 4\varepsilon }{\Delta ^2}. \end{aligned}$$

Expanding and using that $\varepsilon , \Delta < 1$, we obtain

$$\begin{aligned} | \kappa - {\tilde{\kappa }}|\leqslant 2C\Delta ^2 + (3 \kappa + 30 C + 4 )\varepsilon \Delta ^{-2}. \end{aligned}$$

Similarly, in the case $k=3$, we obtain

$$\begin{aligned} | \kappa - {\tilde{\kappa }}|\leqslant 2C'\Delta + (3 \kappa + 14 C' + 4 )\varepsilon \Delta ^{-2}, \end{aligned}$$

where $C'$ is again a constant depending only on $R_{\mathrm{min}},\mathbf{L }$. Since $\kappa \leqslant 1/R_{\mathrm{min}}$, the constants may be chosen to be $A = \max { \lbrace 3/R_{\mathrm{min}}+ 30C + 4, 3/R_{\mathrm{min}}+14C' + 4 \rbrace }$ and $B = \max {\lbrace 2C, 2C' \rbrace }$. They depend only on $R_{\mathrm{min}},\mathbf{L }$.

Now set $\Delta = \varepsilon ^p$ and seek the *p* yielding the fastest rate of convergence of the error bound to zero. Since the exponent in the first term increases with respect to *p* while that in the second decreases, the fastest rate is obtained by requiring the two exponents to be equal, so that $p=1/4$ for $k \geqslant 4$ and $p=1/3$ for $k=3$. $\square$

At the weak feature size the convexity defect function satisfies $h_{\mathbf{M } }(t)=t$. The stability given by item 3. of the properties of $h_{\mathbf{X }}$ stated after Definition 4.1 guarantees that the graph of $h_{{\widetilde{\mathbf{M } }}}$ lies close to that of $h_{\mathbf{M } }$, but this alone cannot be used to approximate the first intersection of the graph of $h_{\mathbf{M } }$ with the diagonal. The graph of $h_{\mathbf{M } }$ could approach the diagonal very slowly before intersecting it, so that the error in approximating an intersection time based on the graph of $h_{{\widetilde{\mathbf{M } }}}$ is not necessarily small.

However, we are only interested in approximating the weak feature size if it yields the reach, i.e., when $R_{\mathrm{wfs}}<R_{\ell }$. Corollary 4.5 guarantees the existence of a discontinuity in $h_{\mathbf{M } }$ at $R_{\mathrm{wfs}}$; in this case the function $h_{\mathbf{M } }$ must jump at $R_{\mathrm{wfs}}$ from being bounded above by a quarter-circle of radius $R_{\ell }$ to intersecting the diagonal. This feature makes it possible to bound the error in an approximation. We begin with a simple lemma.

### Lemma 5.2

Fix $R > 0$. Let the intersection points of the line $y=x-6\varepsilon$ and the quarter-circle $y = R - \sqrt{R^2 - x^2}$ be $(x_0, y_0)$ and $(x_1,y_1)$. Then there is some $\varepsilon _0$, which depends only on *R*, such that for $0< \varepsilon < \varepsilon _0$ the bounds $x_0\leqslant {25}\varepsilon /4$ and $x_1 \geqslant R -{\varepsilon }/{4}$ hold.

### Proof

The equation $x-6\varepsilon = R - \sqrt{R^2 - x^2}$ can be rearranged to give the quadratic one $2 x^2 - (2 R +12 \varepsilon ) x + (36\varepsilon + 12 R ) \varepsilon = 0$ with solutions

$$\begin{aligned} x = \dfrac{2 R +12 \varepsilon \pm \sqrt{(2R - 12\varepsilon )^2 -288\varepsilon ^2 }}{4}. \end{aligned}$$

For sufficiently small values of $\varepsilon$, we have the bound

$$\begin{aligned} 2R -13\varepsilon \leqslant 2R -12\varepsilon -\dfrac{288\varepsilon ^2}{4R-24\varepsilon }\leqslant \sqrt{(2R-12\varepsilon )^2-288\varepsilon ^2}\,, \end{aligned}$$

so that the solutions $x_0$ and $x_1$ are bounded by

$$\begin{aligned} x_0&\leqslant \dfrac{2 R +12\varepsilon - (2R - 13\varepsilon )}{4} = \dfrac{25}{4}\varepsilon ,\\ x_1&\geqslant \dfrac{2 R +12\varepsilon + (2R - 13\varepsilon )}{4} = R -\dfrac{\varepsilon }{4}. \end{aligned}$$

$$\square $$

It is clear from the proof that for any $\delta >0$ there is an $\varepsilon >0$ such that the bounds can be taken to be $(6+\delta )\varepsilon$ and $R_{\ell }-\delta \varepsilon$. It is sufficient to proceed with $\delta = 1/4$ and we will do so.

### Proposition 5.3

Let $\mathbf{M }$ be such that $R(\mathbf{M})>R_{\min }$ and let $\varepsilon <{2}{R}_{\min }/9$ be a positive number small enough that the conclusion of Lemma 5.2 holds for $R=R_{\mathrm{min}}$. Let ${\widetilde{\mathbf{M } }}\subseteq \mathbf{R } ^D$ be such that ${{\,\mathrm{H}\,}}(\mathbf{M } , {\widetilde{\mathbf{M } }}) < \varepsilon$. Now suppose further that $\mathbf{M }$ is such that $R_{\ell }-R_{\mathrm{wfs}}>9\varepsilon /4$. Then the value $r = \inf {\lbrace t \geqslant {22} \varepsilon /4:h_{{\widetilde{\mathbf{M } }}}(t) \geqslant t-3\varepsilon \rbrace }$ satisfies the bound $| R_{\mathrm{wfs}}- r| \leqslant \varepsilon$.

### Proof

We first claim that $r \leqslant R_{\mathrm{wfs}}+ \varepsilon$. To see this, suppose that $R_{\mathrm{wfs}}+ \varepsilon < r$. Then, by the definition of *r*, either $R_{\mathrm{wfs}}+\varepsilon <{22}\varepsilon /4$, which by the assumption on $\varepsilon$ cannot happen, or $h_{{\widetilde{\mathbf{M } }}}(R_{\mathrm{wfs}}+ \varepsilon ) < R_{\mathrm{wfs}}-2\varepsilon$ in which case $R_{\mathrm{wfs}}= h_{\mathbf{M } }(R_{\mathrm{wfs}}) \leqslant h_{{\widetilde{\mathbf{M } }}}(R_{\mathrm{wfs}}+ \varepsilon ) + 2 \varepsilon < R_{\mathrm{wfs}}$, which is a contradiction.

Now let us seek a lower bound for *r*, which relies on the fact that $R=R_{\mathrm{wfs}}$. Note that $h_{\mathbf{M } } (r + \varepsilon ) \geqslant h_{{\widetilde{\mathbf{M } }}} (r) - 2 \varepsilon \geqslant r -5 \varepsilon$. If the additional inequality

$$\begin{aligned} r -5 \varepsilon \geqslant R_{\ell } - \sqrt{R_{\ell }^2 - (r + \varepsilon )^2} \end{aligned}$$

holds, so that $h_{\mathbf{M } } (r + \varepsilon ) > R_{\ell } - \sqrt{R_{\ell }^2 - (r + \varepsilon )^2}$, then by Proposition 4.4 we would have $r + \varepsilon > R = R_{\mathrm{wfs}}$, providing the required lower bound $r \geqslant R_{\mathrm{wfs}}- \varepsilon$ and completing the proof. By Lemma 5.2, this additional inequality holds whenever

$$\begin{aligned} \dfrac{25}{4}\varepsilon \leqslant r + \varepsilon \leqslant R_{\ell } -\dfrac{\varepsilon }{4}. \end{aligned}$$

The first bound is true by the definition of *r*. The second follows from the upper bound for *r* and the gap between $R_{\mathrm{wfs}}$ and $R_{\ell }$: $r \leqslant R_{\mathrm{wfs}}+ \varepsilon \leqslant R_{\ell } - {5}\varepsilon /4$. $\square$

## Minimax Rates for Reach Estimators: Upper Bounds

Every submanifold has a natural uniform probability distribution given by its volume measure. We consider probability distributions with density bounded above and below with respect to this volume measure. Recall the class of manifolds ${\mathfrak {C}}^k_{R_{\mathrm{min}}, \mathbf{L } }$ from [3]: *d*-dimensional compact, connected submanifolds of $\mathbf{R } ^D$ with a lower bound on the reach and admitting a local parametrization with bounded terms in the Taylor expansion (see Definition 3.1).

### Definition 6.1

For $k\geqslant 3$, $R_{\mathrm{min}}>0$, $\mathbf{L }=(L_\perp ,L_3,\ldots ,L_k)$, and $0<f_{\min }\leqslant f_{\max }<\infty$, we let ${\mathscr {P}}^k_{R_{\mathrm{min}},\mathbf{L }}(f_{\min }, f_{\max })$ denote the set of distributions *P* supported on some $\mathbf{M } \in {\mathfrak {C}}^k_{R_{\mathrm{min}}, \mathbf{L } }$ which are absolutely continuous with respect to the volume measure $\mu _{\mathbf{M } }$, with density *f* taking values $\mu _{\mathbf{M } }$-a.s. in $[f_{\min },f_{\max }]$.

This will be abbreviated by ${\mathscr {P}}^k$ where there is no ambiguity. We define the submodels ${\mathscr {P}}^k_\alpha$ to be those distributions supported on elements of ${\mathscr {M}}^k_\alpha$ (the classes defined in Sect. 3). These submodels are such that ${\mathscr {P}}^k=\bigcup _{\alpha \ge 0}{\mathscr {P}}^k_\alpha$. The following lemma shows that the uniform lower bound, $f_{\min }$, on the density of elements of ${\mathscr {P}}^k$ provides an upper bound $R_{\max }$ for both $R_\ell$ and $R_{\mathrm{wfs}}$, which we will use in our estimators later in the section.

### Lemma 6.2

There exists $R_{\max }$ depending on $d, f_{\min }, R_{\mathrm{min}}$ such that, if $P \in {\mathscr {P}}^k$ has support $\mathbf{M}$, then $R_{\ell }, R_{\mathrm{wfs}}\leqslant R_{\max }$.

### Proof

Due to the relationship between curvature and volume, by [4, p. 2, (3)], that $R_\ell \leqslant ({\text {vol}}M / \omega _d)^{1/d} \leqslant (f_{\min } \omega _d)^{-1/d}$, where $\omega _d$ is the volume of the *d*-dimensional sphere of radius 1. Furthermore, Aamari and Levrard have shown [2, Lem. 2.2] that for some constant *C* depending only on dimension, ${{\,\mathrm{diam}\,}}\mathbf{M } \leqslant C(d) f_{\min }^{-1}R_{\mathrm{min}}^{1-d}$. Since $R_{\mathrm{wfs}}\leqslant {{\,\mathrm{diam}\,}}\mathbf{M } /2$ we have $R_{\mathrm{wfs}}\leqslant C(d) f_{\min }^{-1} R_{\mathrm{min}}^{1-d}/2$. Setting

$$\begin{aligned} R_{\max } := \max { \biggl \lbrace (f_{\min } \omega _d)^{-1/d}, \dfrac{C(d) f_{\min }^{-1} R_{\mathrm{min}}^{1-d}}{2}\biggr \rbrace }, \end{aligned}$$

we have the result. $\square$

In [3] the authors construct an estimator ${\widehat{\mathbf{M } }}$ out of polynomial patches, from a sample $(X_1,\ldots , X_n)$ of random variables with common distribution $P \in {\mathscr {P}}^k$, supported on a submanifold $\mathbf{M } \in {\mathfrak {C}}^k_{R_{\mathrm{min}}, \mathbf{L } }$. That estimator has the following convergence property. (Note that the $T_i^*$ referred to below are *i*-linear maps from $T_p \mathbf{M }$ to $\mathbf{R } ^D$ which are the *i*th order terms in the Taylor expansion of the submanifold discussed in Sect. 3.)

### Theorem 6.3

([3, Thm. 6])   Let $k \geqslant 3$. Set

$$\begin{aligned} \theta =\biggl (C_{d,k}\dfrac{f^2_{\max }\log n}{(n-1) f^3_{\min }}\biggr )^{\!1/d} \end{aligned}$$

for $C_{d,k}$ large enough. If *n* is large enough so that $0 < \theta \leqslant \min {\lbrace R_{\min }, L^{-1}_{\perp }\rbrace }/8$ and $\theta ^{-1} \geqslant C_{d,k,R_{\min },\mathbf{L } } \geqslant \sup _{2 \leqslant i \leqslant k}|T_i^*|_{op }$, then with probability at least $1 - 2({1}/{n})^{{k}/{d}}$, we have

$$\begin{aligned} {{\,\mathrm{H}\,}}({\widehat{\mathbf{M } }}, \mathbf{M } ) \leqslant C^\star \, \theta ^k \end{aligned}$$

for some $C^\star >0$. In particular, for *n* large enough,

$$\begin{aligned} \sup _{P \in {\mathscr {P}}^k} \mathbf{E } _{P^{\otimes n}} \bigl [{{\,\mathrm{H}\,}}({\widehat{\mathbf{M } }}, \mathbf{M } )\bigr ] \leqslant C \biggl ( \dfrac{\log n}{n-1} \biggr ) ^{\!k/d}, \end{aligned}$$

where $$C = C_{d,k,R_{\min },\mathbf{L } ,f_{\min },f_{\max }}$$.

Note that the estimator is dependent on the value of $\theta \approx n^{-1/d}$ to within logarithmic terms, which serves as a bandwidth. The convergence rate of this estimator is very close to the currently established lower bound for estimating the reach *R*, which is $n^{-k/d}$; see Theorem 7.1 in Sect. 7 below.

### Estimating the Local Reach

#### Definition 6.4

We define an estimator for $R_{\ell }(\mathbf{M } )$, the local reach of a submanifold $\mathbf{M }$, by

$$\begin{aligned} {\widehat{R}}_{\ell } = \min {\biggl \{\dfrac{\Delta ^2}{2h_{{\widehat{\mathbf{M } }}}(\Delta )}, R_{\max }\biggr \}}, \end{aligned}$$

where ${\widehat{\mathbf{M } }}$ is the Aamari–Levrard estimator of $\mathbf{M}$ as discussed at the beginning of Sect. 6 above, $\varepsilon = C^\star \theta ^k$ as in Theorem 6.3, $\Delta =\varepsilon ^{1/3}$ if $k=3$ or $\Delta =\varepsilon ^{1/4}$ if $k \geqslant 4$, and $R_{\max }$ is as in Lemma 6.2.

#### Theorem 6.5

Let $k \geqslant 3$, let $\theta$ be as in Theorem 6.3, and set $\varepsilon = C^\star \theta ^k$. Then with probability at least $1 - 2 ({1}/{n})^{{k}/{d}}$, we have

$$\begin{aligned} | {\widehat{R}}_{\ell } -R_\ell | \leqslant C_{d,k,R_{\min },\mathbf{L } ,f_{\min }} \varepsilon ^{1/3}, \end{aligned}$$

and, where $k \geqslant 4$, the exponent is $\varepsilon ^{1/2}$. Moreover, for *n* large enough, we have

$$\begin{aligned} \sup _{P \in {\mathscr {P}}^k} \mathbf{{E}}_{P^{\otimes n}}[| {\widehat{R}}_{\ell } -R_\ell |] \leqslant C \biggl ( \dfrac{\log n}{n-1} \biggr )^{\!{k}/({3d})}, \end{aligned}$$

or, for $k \geqslant 4$, $C(\log n/{(n-1)})^{{k}/({2d})}$, where $C = C_{d,k,R_{\min },\mathbf{L } ,f_{\min },f_{\max }}$.

A glance at the proof shows that we actually control $\bigl |{{\widehat{R}}_{\ell }}^{-1}-{R_\ell }^{-1}\bigr |$ rather than $|{\widehat{R}}_{\ell } - R_\ell |$. This has no impact since $R_\ell \leqslant R_{\max }$ is uniformly bounded and we do not seek fine control on *C*. Changing the parametrization $R \mapsto 1/R$ in our statistical problem and estimating 1/*R* instead of *R* would enable us to remove the projection onto $[0, R_{\max }]$ that we use to define ${\widehat{R}}_{\ell }$.

#### Proof

By construction, ${\widehat{R}}_{\ell } \leqslant R_{\max }$, and it is also clear that

$$\begin{aligned} \biggl |\dfrac{1}{{\widehat{R}}_{\ell }}-\dfrac{1}{R_\ell }\biggr | \leqslant \biggl |2\dfrac{h_{{\widehat{\mathbf{M } }}}(\Delta )}{\Delta ^2}-\dfrac{1}{R_\ell }\biggr |. \end{aligned}$$

We derive

$$\begin{aligned} |{\widehat{R}}_{\ell } - R_\ell |={\widehat{R}}_{\ell }R_\ell \biggl |\dfrac{1}{{\widehat{R}}_{\ell }}-\dfrac{1}{R_\ell }\biggr |\leqslant R_{\max }^2 \biggl |2\dfrac{h_{{\widehat{\mathbf{M } }}}(\Delta )}{\Delta ^2}-\dfrac{1}{R_\ell }\biggr |. \end{aligned}$$

The first statement of Theorem 6.5 is then a straightforward consequence of Proposition 5.1 together with Theorem 6.3. Next, we have

$$\begin{aligned}&\mathbf{E }_{P^{\otimes n}}[|{\widehat{R}}_{\ell } - R_\ell |] \\&\quad \leqslant C_{d,k,R_{\min },f_{\min }, \mathbf{L }} \varepsilon ^{1/3} + 2R_{\max } P^{\otimes n}\bigl (|{\widehat{R}}_{\ell } - R_\ell | > C_{d,k,R_{\min },f_{\min }, \mathbf{L }} \varepsilon ^{1/3}\bigr ) \\&\quad \leqslant C_{d,k,R_{\min },f_{\min }, \mathbf{L }} \varepsilon ^{1/3} +4 R_{\max } n^{-k/d}, \end{aligned}$$

thanks to the first part of Theorem 6.5. This term is of order $(\log n/n)^{k/3d}$. For $k \geqslant 4$, we have the improvement to the exponent $\varepsilon ^{1/2}$ and the order becomes $(\log n/n)^{k/2d}$, which establishes the second part of the theorem for all values of $k \geqslant 3$ and completes the proof. $\square$

For $k=3,4$, then, the constructed estimator is optimal up to a $\log n$ factor as follows from Theorem 7.1 below.

### Estimating the Global Reach

By the earlier discussion, it is not possible to give a convergence guarantee when estimating the weak feature size, i.e., the first positive critical value of $d_{\mathbf{M } }$. However, in the case where $R = R_{\mathrm{wfs}}$, that is, when $R_{\mathrm{wfs}}< R_{\ell }$, this is possible. Accordingly, we now define an estimator for $R_{\mathrm{wfs}}$ and hence an estimator for the reach itself.

#### Definition 6.6

We define an estimator for $R_{\mathrm{wfs}}$, the weak feature size of a submanifold $\mathbf{M }$, by

$$\begin{aligned} {\widehat{R}}_{\text {wfs}} = \min {\bigl \{\inf {\lbrace t \in \mathbf{R } :{22}\varepsilon <4t,\, h_{{\widehat{\mathbf{M } }}}(t) \geqslant t - 3 \varepsilon \rbrace },\,R_{\max }\bigr \}}, \end{aligned}$$

where ${\widehat{\mathbf{M } }}$ is the Aamari–Levrard estimator of $\mathbf{M}$ as discussed at the beginning of Sect. 6 above, $\varepsilon =C^\star \theta ^k$ as in Theorem 6.3, and $R_{\max }$ is as in Lemma 6.2.

Our estimator for the reach is then the lesser of the two individual estimators.

#### Definition 6.7

Let $C^\star , \theta$ be as in Theorem 6.3 and set $\varepsilon = C^\star \theta ^k$. We define an estimator for $R(\mathbf{M } )$, the reach of a submanifold $\mathbf{M }$, by

$$\begin{aligned} \widehat{R} = \min {\{{\widehat{R}}_{\text {wfs}}, \widehat{R}_{\ell }\}}. \end{aligned}$$

Note that we could just as well use ${\widehat{R}}_{\ell }$ in place of $R_{\max }$ to cap the value of ${\widehat{R}}_{\text {wfs}}$, since we do not analyze the error in the case ${\widehat{R}}_{\ell } < {\widehat{R}}_{\text {wfs}}$. However, Definition 6.6 appears more natural as a stand-alone estimator of $R_{\mathrm{wfs}}$.

#### Theorem 6.8

Let $k \geqslant 3$, let $C^\star ,\theta$ be as in Theorem 6.3, and set $\varepsilon = C^\star \theta ^k$, with $\varepsilon$ such that ${22}\varepsilon /4<\min {\{R_{\min },1\}}$, which is always satisfied for large enough $n\geqslant 1$. Then with probability at least $1-4n^{-k/d}$, we have

$$\begin{aligned} | \widehat{R} - R| \leqslant C_{d,k,R_{\min },\mathbf{L } } \varepsilon ^{1/3}, \end{aligned}$$

and, where $k \geqslant 4$, the exponent is $\varepsilon ^{1/2}$. In particular, for *n* large enough,

$$\begin{aligned} \sup _{P \in {\mathscr {P}}^k} \mathbf{E } _{P^{\otimes n}} [| \widehat{R} - R|] \leqslant C \biggl ( \dfrac{\log n}{n-1} \biggr )^{\!{k}/({3d})}, \end{aligned}$$

or, for $k \geqslant 4$, $C({\log n}/({n-1}))^{{k}/({2d})}$, where $C = C_{d,k,\tau _{\min },\mathbf{L } ,f_{\min },f_{\max }}$.

#### Proof

We will prove the result in three steps. In Step 1 we provide a bound in the case ${\widehat{R}}_\ell < {\widehat{R}}_{\text {wfs}}$ which holds with high probability. Then in Step 2 we provide a bound in the complementary case ${\widehat{R}}_\ell \geqslant {\widehat{R}}_{\text {wfs}}$. Finally, in Step 3, we combine the two bounds, proving the first statement, and use it to obtain the bound on the expected loss. In the following, we use the letters *C* and $C'$ to denote positive numbers that do not depend on *n* and that may vary at each occurrence.

*Step 1*. We have

$$\begin{aligned} |{\widehat{R}}-R|\mathbf{1} _{\{{\widehat{R}}_\ell< {\widehat{R}}_{\text {wfs}}\}}&=|{\widehat{R}}_\ell -\min {\{R_\ell ,R_{\text {wfs}}\}}|\mathbf{1} _{\{{\widehat{R}}_\ell< {\widehat{R}}_{\text {wfs}}\}} \\&\leqslant |{\widehat{R}}_\ell -R_\ell | +|{\widehat{R}}_\ell -R_{\text {wfs}}|\mathbf{1} _{(R_{\text {wfs}}< R_\ell )}{} \mathbf{1} _{\{{\widehat{R}}_\ell< {\widehat{R}}_{\text {wfs}}\}} \\&\leqslant 2|{\widehat{R}}_\ell -R_\ell | + |R_\ell -R_{\text {wfs}}|\mathbf{1} _{(R_{\text {wfs}}< R_\ell )}{} \mathbf{1} _{\{{\widehat{R}}_\ell < {\widehat{R}}_{\text {wfs}}\}} \end{aligned}$$

by the triangle inequality. For $C_1,C_2 >0$, introduce the events

$$\begin{aligned} \Omega _1 = \{|{\widehat{R}}_\ell -R_\ell | \leqslant C_1\varepsilon ^{1/3}\}\quad \;\;\text {and}\quad \;\;\Omega _2 = \bigl \{{{\,\mathrm{H}\,}}({\widehat{\mathbf{M } }},\mathbf{M } ) \leqslant \varepsilon \bigr \}. \end{aligned}$$

On $\{{\widehat{R}}_\ell < {\widehat{R}}_{\text {wfs}}\}$, we have

1 $$\begin{aligned} h_{{\widehat{\mathbf{M } }}}(t) < t-3\varepsilon \end{aligned}$$

for all $t \in [{22}\varepsilon /4, {\widehat{R}}_\ell ]$, therefore, on $\{{\widehat{R}}_\ell < {\widehat{R}}_{\text {wfs}}\} \cap \Omega _1$, we infer that (1) holds for $t \in [{22}\varepsilon /4, R_\ell -C_1\varepsilon ^{1/3}]$. By 3. of the properties of the convexity defect function given after Definition 4.1, on $\Omega _2$ we have

$$\begin{aligned} h_{{\widehat{\mathbf{M } }}}(t) \geqslant h_{\mathbf{M } }(t-\varepsilon )-2\varepsilon . \end{aligned}$$

Putting the last two estimates together, we obtain on $\{{\widehat{R}}_\ell < {\widehat{R}}_{\text {wfs}}\} \cap \Omega _1 \cap \Omega _2$ the bound

$$\begin{aligned} h_{\mathbf{M } }(t-\varepsilon ) < t - 3\varepsilon + 2\varepsilon \end{aligned}$$

for all $t \in [{22}\varepsilon /4, R_\ell -C_1\varepsilon ^{1/3}]$, or, equivalently, $h_{\mathbf{M } }(t) < t$ for $t \in [({22}/{4}-1)\varepsilon , R_\ell -C_1\varepsilon ^{1/3}-\varepsilon ]$. Therefore $h_{\mathbf{M } }(t)<t$ for $t \leqslant R_\ell -C_1\varepsilon ^{1/3}-\varepsilon$ and this implies in turn

$$\begin{aligned} R_{\text {wfs}} \geqslant R_\ell -C_1\varepsilon ^{1/3}-\varepsilon . \end{aligned}$$

We have thus proven

$$\begin{aligned} |R_\ell -R_{\text {wfs}}|\mathbf{1} _{\{R_{\text {wfs}}< R_\ell \}}{} \mathbf{1} _{\{{\widehat{R}}_\ell < {\widehat{R}}_{\text {wfs}}\}}{} \mathbf{1} _{\Omega _1\cap \Omega _2} \leqslant (C_1\varepsilon ^{1/3}+\varepsilon ) \leqslant C\varepsilon ^{1/3}. \end{aligned}$$

Finally

$$\begin{aligned} |{\widehat{R}}-R|\mathbf{1} _{\{{\widehat{R}}_\ell < {\widehat{R}}_{\text {wfs}}\}}{} \mathbf{1} _{\Omega _1\cap \Omega _2} \leqslant C\varepsilon ^{1/3}. \end{aligned}$$

*Step 2*. We have

$$\begin{aligned} |{\widehat{R}}-R|\mathbf{1} _{\{{\widehat{R}}_\ell \geqslant {\widehat{R}}_{\text {wfs}}\}}\leqslant T_1+T_2+T_3, \end{aligned}$$

with

$$\begin{aligned} T_1&= |{\widehat{R}}_{\text {wfs}}-R_{\text {wfs}}|\mathbf{1} _{\{R_{\text {wfs}}+{9}\varepsilon /4< R_\ell \}}{} \mathbf{1} _{\{{\widehat{R}}_\ell \geqslant {\widehat{R}}_{\text {wfs}}\}}, \\ T_2&= |{\widehat{R}}_{\text {wfs}}-R_{\text {wfs}}|\mathbf{1} _{\{R_{\text {wfs}} \leqslant R_\ell< R_{\text {wfs}}+{9}\varepsilon /4\}}{} \mathbf{1} _{\{{\widehat{R}}_\ell \geqslant {\widehat{R}}_{\text {wfs}}\}}, \\ T_3&=|{\widehat{R}}_{\text {wfs}}- R_\ell |\mathbf{1} _{\{R_\ell < R_{\text {wfs}}\}}{} \mathbf{1} _{\{{\widehat{R}}_\ell \geqslant {\widehat{R}}_{\text {wfs}}\}}. \end{aligned}$$

By Proposition 5.3, we have $T_1 \leqslant \varepsilon$ on $\Omega _2$. We turn to the term $T_2$. We have

$$\begin{aligned} h_{{\widehat{\mathbf{M } }}}({\widehat{R}}_{\text {wfs}}) \geqslant {\widehat{R}}_{\text {wfs}} - 3\varepsilon \end{aligned}$$

on $\{{\widehat{R}}_\ell \geqslant {\widehat{R}}_{\text {wfs}}\}$ by construction. Thanks to item 3. of the properties of the convexity defect function given after Definition 4.1, we also have

$$\begin{aligned} h_{{\widehat{\mathbf{M } }}}({\widehat{R}}_{\text {wfs}}) \leqslant h_{\mathbf{M } }({\widehat{R}}_{\text {wfs}} + \varepsilon )+2\varepsilon \end{aligned}$$

on $$\Omega _2$$. Therefore,

$$\begin{aligned} {\widehat{R}}_{\text {wfs}}-5\varepsilon \leqslant h_{\mathbf{M } }({\widehat{R}}_{\text {wfs}} + \varepsilon ) \end{aligned}$$

holds true on $\{{\widehat{R}}_\ell \geqslant {\widehat{R}}_{\text {wfs}}\} \cap \Omega _2$. Introduce now the event

$$\begin{aligned} \Omega _3 = \{{\widehat{R}}_{\text {wfs}}+\varepsilon < R_{\text {wfs}}\}. \end{aligned}$$

By Proposition 4.4, it follows that

$$\begin{aligned} {\widehat{R}}_{\text {wfs}} - 5\varepsilon \leqslant R_\ell -\sqrt{R_\ell ^2-({\widehat{R}}_{\text {wfs}}+\varepsilon )^2} \end{aligned}$$

on $\{{\widehat{R}}_\ell \geqslant {\widehat{R}}_{\text {wfs}}\} \cap \Omega _2 \cap \Omega _3$. Solving this inequality when $R_\ell > {\widehat{R}}_{\text {wfs}}+\varepsilon$ yields ${\widehat{R}}_{\text {wfs}} \geqslant R_\ell -C\varepsilon$ for some $C>0$ that depends on $R_\ell$ only. Otherwise, $R_\ell -\varepsilon \leqslant {\widehat{R}}_{\text {wfs}}$ directly. Replacing *C* by $\max {\lbrace 1,C \rbrace }$, we infer

$$\begin{aligned} R_\ell {-C\varepsilon }\leqslant {\widehat{R}}_{\text {wfs}} \leqslant {\widehat{R}}_\ell \leqslant R_\ell + C_1\varepsilon ^{1/3} \end{aligned}$$

on $\{{\widehat{R}}_\ell \geqslant {\widehat{R}}_{\text {wfs}}\} \cap \Omega _1 \cap \Omega _2 \cap \Omega _3$ hence $|{\widehat{R}}_{\text {wfs}}-R_\ell | \leqslant {C}\varepsilon ^{1/3}$ on that event. Combining this estimate with the condition $|R_\ell - R_{\text {wfs}}| \leqslant {9}\varepsilon /4$ in the definition of $T_2$ implies

$$\begin{aligned} |{\widehat{R}}_{\text {wfs}}-R_{\text {wfs}}| \leqslant {C}\varepsilon ^{1/3}+\dfrac{9}{4}\varepsilon . \end{aligned}$$

We have thus proven

$$\begin{aligned} T_2 \mathbf{1} _{\bigcap _{i = 1}^3\Omega _i} \leqslant {C}\varepsilon ^{1/3}+\dfrac{9}{4}\varepsilon \leqslant {C'}\varepsilon ^{1/3}. \end{aligned}$$

On the complementary event $\Omega _3^c = \{{\widehat{R}}_{\text {wfs}}+\varepsilon \geqslant R_{\text {wfs}}\}$, we have, on the one hand,

$$\begin{aligned} R_{\text {wfs}} - {\widehat{R}}_{\text {wfs}} \leqslant \varepsilon . \end{aligned}$$

But on the other hand, on $\{{\widehat{R}}_\ell \geqslant {\widehat{R}}_{\text {wfs}}\} \cap \Omega _1$ we have

$$\begin{aligned} {\widehat{R}}_{\text {wfs}}-R_{\text {wfs}} \leqslant {\widehat{R}}_\ell - R_{\text {wfs}} \leqslant R_\ell - R_{\text {wfs}} + C_1\varepsilon ^{1/3} \leqslant \dfrac{9}{4}\varepsilon + C_1\varepsilon ^{1/3} \leqslant {C}\varepsilon ^{1/3} \end{aligned}$$

thanks to the condition $|R_\ell - R_{\text {wfs}}| \leqslant {9}\varepsilon /4$ in the definition of $T_2$. Combining these bounds, we obtain

$$\begin{aligned} T_2 (1-\mathbf{1} _{\Omega _3})\mathbf{1} _{\Omega _1} \leqslant {C}\varepsilon ^{1/3}. \end{aligned}$$

Putting together this estimate and the bound $T_2\mathbf{1} _{\bigcap _{i = 1}^3\Omega _i} \leqslant {C}\varepsilon ^{1/3}$ we established previously, we derive

$$\begin{aligned} T_2\mathbf{1} _{\Omega _1 \cap \Omega _2} \leqslant {C}\varepsilon ^{1/3}. \end{aligned}$$

We finally turn to the term $T_3$. On $\{{\widehat{R}}_{\text {wfs}} \geqslant R_\ell \}$ intersected with $\{{\widehat{R}}_\ell \geqslant {\widehat{R}}_{\text {wfs}}\} \cap \Omega _1$, we have

$$\begin{aligned} 0 < R_\ell \leqslant {\widehat{R}}_{\text {wfs}} \leqslant {\widehat{R}}_\ell \leqslant R_\ell + C_1\varepsilon ^{1/3}, \end{aligned}$$

which yields the estimate

$$\begin{aligned} | {\widehat{R}}_{\text {wfs}} - R_\ell | \leqslant C_1\varepsilon ^{1/3}\quad \text {on}\ \ \{{\widehat{R}}_{\text {wfs}} \geqslant R_\ell \} \cap \{{\widehat{R}}_\ell \geqslant {\widehat{R}}_{\text {wfs}}\} \cap \Omega _1. \end{aligned}$$

Alternatively, on the complementary event $\{{\widehat{R}}_{\text {wfs}} < R_\ell \}$ intersected with $\Omega _2\cap$ $\{{\widehat{R}}_\ell \geqslant {\widehat{R}}_{\text {wfs}}\}$ we have ${\widehat{R}}_{\text {wfs}} - 5\varepsilon \leqslant R_\ell -\sqrt{R_\ell ^2-({\widehat{R}}_{\text {wfs}}+\varepsilon )^2}$ in the same way as for the term $T_2$, provided ${\widehat{R}}_{\text {wfs}}+\varepsilon < R_\ell$. This implies ${\widehat{R}}_{\text {wfs}} \geqslant R_\ell {-C\varepsilon }$. Otherwise ${\widehat{R}}_{\text {wfs}}+\varepsilon \geqslant R_\ell$ holds true. In any event, we obtain $- {C}\varepsilon \leqslant {\widehat{R}}_{\text {wfs}} -R_\ell$. Since ${\widehat{R}}_{\text {wfs}} -R_\ell \leqslant C_1\varepsilon ^{1/3}$ on $\Omega _1$, we conclude

$$\begin{aligned} | {\widehat{R}}_{\text {wfs}} -R_\ell | \leqslant \varepsilon + C_1\varepsilon ^{1/3} \leqslant C\varepsilon ^{1/3}\quad \text {on}\ \ \{{\widehat{R}}_{\text {wfs}} < R_\ell \} \cap \{{\widehat{R}}_\ell \geqslant {\widehat{R}}_{\text {wfs}}\} \cap \Omega _1 \cap \Omega _2. \end{aligned}$$

Combining these two bounds for $| {\widehat{R}}_{\text {wfs}} -R_\ell |$, we finally derive

$$\begin{aligned} T_3 \mathbf{1} _{\Omega _1 \cap \Omega _2} \leqslant C\varepsilon ^{1/3}. \end{aligned}$$

Putting together our successive estimates for $T_1$, $T_2$, and $T_3$, we have proven

$$\begin{aligned} |{\widehat{R}}-R|\mathbf{1} _{\{{\widehat{R}}_\ell \geqslant {\widehat{R}}_{\text {wfs}}\}}{} \mathbf{1} _{\Omega _1 \cap \Omega _2} \leqslant \varepsilon + 2C\varepsilon ^{1/3}\leqslant C'\varepsilon ^{1/3}. \end{aligned}$$

*Step 3.* Combining Steps 1 and 2 yields

$$\begin{aligned} |{\widehat{R}}-R|\mathbf{1} _{\Omega _1 \cap \Omega _2} \leqslant C\varepsilon ^{1/3}. \end{aligned}$$

By Theorem 6.5, we have $P^{\otimes n}(\Omega _1) \geqslant 1-2n^{-k/d}$ as soon as $C_1 \geqslant C_{d,k,R_{\min },f_{\min },\mathbf{L }}$. By Theorem 6.3, we have $P^{\otimes n}(\Omega _2) \geqslant 1-2n^{-k/d}$. The first estimate in Theorem 6.8 follows for $k \geqslant 3$. The improvement in the case $k=4$ is done in exactly the same way and we omit it. Finally, integrating, we obtain

$$\begin{aligned} \mathbf{E }_{P^{\otimes n}}[|{\widehat{R}}-R|]&\leqslant C\varepsilon ^{1/3}+2R_{\max } \bigl (P^{\otimes n}(\Omega _1^c)+ P^{\otimes n}(\Omega _2^c)\bigr ) \\&\leqslant C\varepsilon ^{1/3}+4R_{\max }n^{-k/d} \leqslant C'\varepsilon ^{1/3} \end{aligned}$$

and the second statement of Theorem 6.8 is proven for $k \geqslant 3$. The improvement in the case $k=4$ follows in similar fashion. $\square$

## Minimax Rates for Reach Estimators: Lower Bounds

We fix $R_{\min }$, $\mathbf{L }$, *k*, $f_{\min }$, and $f_{\max }$, and recall the classes ${\mathscr {P}}^k_\alpha$ which were defined in Sect. 6, parametrized by the gap $\alpha \leqslant R_{\ell } - R_{\mathrm{wfs}}$. These sub-models are such that ${\mathscr {P}}^k = \bigcup _{\alpha \geqslant 0} {\mathscr {P}}^k_\alpha$.

### Theorem 7.1

If $f_{\min }$ is small enough and $f_{\max }$, $\mathbf{L }$ are large enough (depending on $R_{\min }$, and on $\alpha$ for the second statement), then we have the following lower bounds on the reach estimation problem:

$$\begin{aligned} \liminf _{n\rightarrow \infty }\, n^{(k-2)/d} \inf _{{\widehat{R}}} \sup _{P \in {\mathscr {P}}^k_0} \mathbf{E}_{P^{\otimes n}}[|{\widehat{R}} - R|]&\geqslant C_0> 0\qquad \text {and}\\ \liminf _{n\rightarrow \infty }\, n^{k/d} \inf _{{\widehat{R}}} \sup _{P \in {\mathscr {P}}^k_\alpha } \mathbf{E}_{P^{\otimes n}}[|{\widehat{R}} - R|]&\geqslant C_{\alpha }> 0\qquad \text {for all }\alpha > 0, \end{aligned}$$

with $C_0$ depending on $R_{\min }$ and $C_{\alpha }$ depending on $R_{\min }$ and $\alpha$.

In particular, the minimax rate on the whole model ${\mathscr {P}}^k$ is of order $n^{-({k-2})/{d}}$. To show this proposition, we will make use of Le Cam’s Lemma, restated in our context.

### Lemma 7.2

(Le Cam’s Lemma, [18]) For any two $P_1, P_2 \in {\mathscr {P}}$, where ${\mathscr {P}}$ is a model of manifold-supported probability measures, we have

$$\begin{aligned} \inf _{{\widehat{R}}} \sup _{P \in {\mathscr {P}}} \mathbf{E}_{P^{\otimes n}}[|{\widehat{R}} - R|] \geqslant \dfrac{|R_1 - R_2|}{2} (1 - {\text {TV}}(P_1,P_2))^n, \end{aligned}$$

where ${\text {TV}}$ denotes the total variation distance between measures and $R_1$ (respectively $R_2$) denotes the reach of the support of $P_1$ (resp. $P_2$).

Therefore, one needs to compute the total variation distance between two given manifold-supported measures. When these measures are uniform over their support, we have the following convenient formula.

### Lemma 7.3

Let $M_1,M_2$ be two compact *d*-dimensional submanifolds of $\mathbf{R}^D$ and let $P_1,P_2$ be the uniform distributions over $M_1$ and $M_2$. Then we have

$$\begin{aligned} {\text {TV}}(P_1,P_2) = \dfrac{{\mathscr {H}}^d(M_2 \setminus M_1)}{{\text {vol}}M_2} \end{aligned}$$

if ${\text {vol}}M_2 \geqslant {\text {vol}}M_1$, where ${\mathscr {H}}^d$ denotes the *d*-dimensional Hausdorff measure on ${\mathbf{R}}^D$.

### Proof

First note that $P_1$ and $P_2$ are absolutely continuous with respect to ${\mathscr {H}}^d$ with densities $\mathbf{1}_{M_1}/({{\text {vol}}M_1})$ and $\mathbf{1}_{M_2}/({{\text {vol}}M_2})$ respectively. Therefore, we have the following chain of equalities:

$$\begin{aligned} {\text {TV}}(P_1,P_2)&= \dfrac{1}{2} \int \, \biggl |\dfrac{\mathbf{1}_{M_1} }{{\text {vol}}M_1} - \dfrac{\mathbf{1}_{M_2} }{{\text {vol}}M_2} \biggr |\, \mathrm{d}{\mathscr {H}}^d \\&= \dfrac{{\mathscr {H}}^d(M_1 \setminus M_2)}{2{\text {vol}}M_1} + \dfrac{{\mathscr {H}}^d(M_2 \setminus M_1)}{2{\text {vol}}M_2} \\&\quad + \dfrac{{\mathscr {H}}^d(M_1 \cap M_2)}{2} \biggl ( \dfrac{1}{{\text {vol}}M_1} - \dfrac{1}{{\text {vol}}M_2} \biggr ) \\&= \dfrac{1}{2} \left\{ 1 + \dfrac{{\mathscr {H}}^d(M_2 \setminus M_1) - {\mathscr {H}}^d(M_1 \cap M_2)}{{\text {vol}}M_2}\right\} = \dfrac{{\mathscr {H}}^d(M_2 \setminus M_1)}{{\text {vol}}M_2}. \end{aligned}$$

$$\square $$

Before proving Theorem 7.1 we need to introduce the following technical result:

### Lemma 7.4

Let $\Phi :\mathbf{R}^d \rightarrow \mathbf{R}$ be a smooth function and $M \!=\! \{(v,\Phi (v)):v \!\in \! \mathbf{R}^d\} \subseteq \mathbf{R}^{d+1}$ be its graph. The second fundamental form of *M* at the point $x = (v,\Phi (v)) \in M$ is given by

$$\begin{aligned} \mathrm{I\,\!I}_x(u,w) = \dfrac{\mathrm{d}^2 \Phi (v)[{{\,\mathrm{pr}\,}}u,{{\,\mathrm{pr}\,}}w]}{\sqrt{1 + \Vert \nabla \Phi (v)\Vert ^2}}\quad \text { for all }\ u,w \in T_x M \end{aligned}$$

where ${{\,\mathrm{pr}\,}}$ is the linear projection to $\mathbf{R}^d \subseteq \mathbf{R}^{d+1}$.

### Proof

We define $\Psi :v \in {\mathbf{R }}^d \mapsto (v,\Phi (v)) \in {\mathbf{R }}^{d+1}$ so that *M* is the image of ${\mathbf{R }}^d$ through the diffeomorphism $\Psi$. Let $x\in M$ and let $v \in {\mathbf{R }}^d$ be such that $x = \Psi (v)$. The tangent space $T_x M$ is given by $T_x M = \{ \mathrm{d}\Psi (v)[h] = (h, \langle h, \nabla \Phi (v) \rangle ):h \in {\mathbf{R }}^d\}$, so that a normal vector field on *M* is given by

$$\begin{aligned} n(x) = \Biggl ( -\dfrac{\nabla \Phi (v)}{\sqrt{1 + \Vert \nabla \Phi (v)\Vert ^2}}, \dfrac{1}{\sqrt{1 + \Vert \nabla \Phi (v)\Vert ^2}}\Biggr ) \in {\mathbf{R }}^{d+1}. \end{aligned}$$

For $u \in T_x M$, where $h = {{\,\mathrm{pr}\,}}u$, we have

$$\begin{aligned} \mathrm{d}n(x)[u] = \Biggl ( - \dfrac{H\Phi (v) h}{\sqrt{1 + \Vert \nabla \Phi (v)\Vert ^2}}, 0\Biggr ) - \dfrac{\langle H\Phi (v) h, \nabla \Phi (v) \rangle }{1 + \Vert \nabla \Phi (v)\Vert ^2} n(x) , \end{aligned}$$

where $H\Phi$ denotes the Hessian of $\Phi$. Now for $w \in T_x M$ and $\eta = {{\,\mathrm{pr}\,}}w$, we have

$$\begin{aligned} \mathrm{I\,\!I}_x(u,w)&= - \langle \mathrm{d}n(x)[u], w \rangle = \Biggl \langle \Biggl ( \dfrac{H\Phi (v) h}{\sqrt{1 + \Vert \nabla \Phi (v)\Vert ^2}}, 0\Biggr ), (\eta , \langle \eta , \nabla \Phi (v) \rangle ) \Biggr \rangle \\&=\Biggl \langle \dfrac{H\Phi (v)h}{\sqrt{1 + \Vert \nabla \Phi (v)\Vert ^2}}, \eta \Biggr \rangle = \dfrac{\mathrm{d}^2 \Phi (v)[h,\eta ]}{\sqrt{1 + \Vert \nabla \Phi (v)\Vert ^2}} \end{aligned}$$

concluding the proof. $\square$

We are now ready to prove Theorem 7.1.

### Proof of Theorem 7.1

*Step 1: The case of* ${\mathscr {P}}^k_0$. Let *M* be the *d*-dimensional sphere in ${\mathbf{R }}^{d+1}$ of radius *r* centered at $- r e_{d+1}$, where $e_{d+1} = (0, \ldots , 0, 1)$. We choose *r* to be such that $r \geqslant 2R_{\min }$. Since *M* is smooth, there exists $\mathbf{L } ^* \in {\mathbf{R }}^{k-2}$ (depending on *r*) such that $M \in {\mathfrak {C}}^k_{r,\mathbf{L } ^*}$, and thus the uniform probability *P* on *M* is in ${\mathscr {P}}^k_{r,\mathbf{L } ^*}(a^*,a^*)$ (see Definition 6.1) with $a^* = (r^d s_d)^{-1}$ and $s_d$ being the volume of the unit *d*-dimensional sphere.

**Fig. 3:**
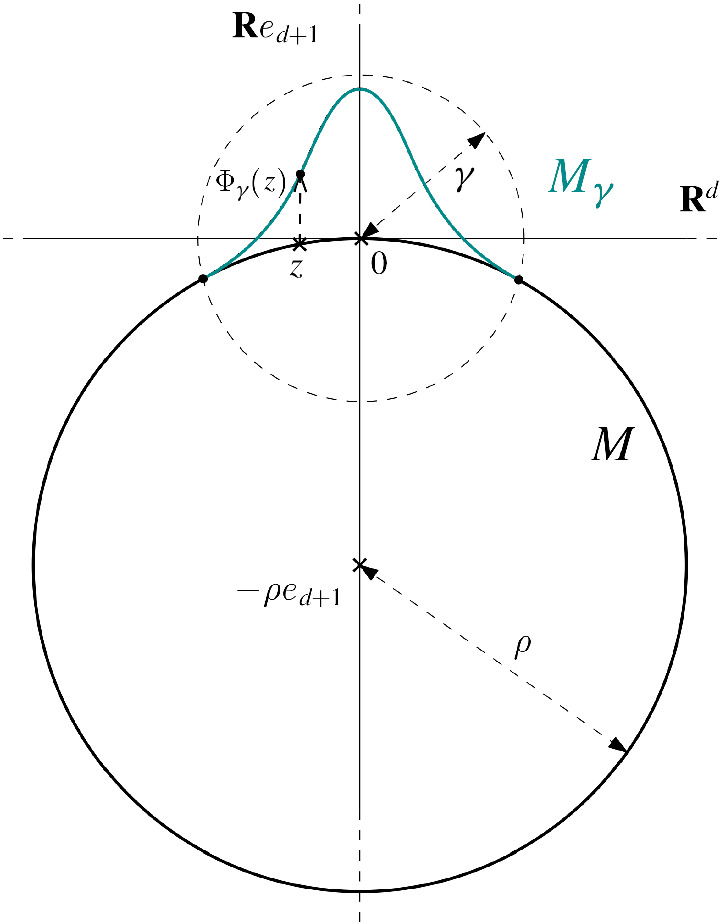
The submanifolds *M* and $M_\gamma$ used in the first part of the proof of the lower bound

Let us now perturb *M* to $M_\gamma$, as illustrated in Fig. 3. Define for any $\gamma > 0$

$$\begin{aligned} \Phi _\gamma :{\left\{ \begin{array}{ll} {\mathbf{R }}^{d+1} \rightarrow {\mathbf{R }}^{d+1}, \\ z \mapsto z + \gamma ^k \Psi (z/\gamma ) e_{d+1}, \end{array}\right. } \end{aligned}$$

where $\Psi (z) = \psi (\Vert z\Vert )$ and where $\psi :{\mathbf{R }}\rightarrow {\mathbf{R }}$ is a smooth, even, non-trivial, positive map supported on $[-1,1]$, decreasing on [0, 1], and with $\psi ''(0) < 0$. The above map is a global diffeomorphism as soon as $\gamma ^{k-1} \Vert \mathrm{d}\Psi \Vert _{{{\,\mathrm{op}\,}},\infty } < 1$. Moreover, we have $\Vert \mathrm{d}\Phi _\gamma -I_D\Vert _{{{\,\mathrm{op}\,}},\infty } = \gamma ^{k-1}\Vert \mathrm{d}\Psi \Vert _{{{\,\mathrm{op}\,}},\infty }$ and $\Vert \mathrm{d}^j \Phi _\gamma \Vert _{{{\,\mathrm{op}\,}},\infty } \leqslant \gamma ^{k-j} \Vert \mathrm{d}^j\Psi \Vert$, so that, provided $\Vert \mathrm{d}^k \Psi \Vert$ is chosen small enough (depending on *r*) and that $\gamma$ is small enough (depending again on *r*), then we can apply Proposition A.5 from the supplementary material in [3] to show that the submanifold $M_\gamma = \Phi _\gamma (M)$ is in ${\mathfrak {C}}^k_{r/2, 2\mathbf{L } ^*}$. Then we have

$$\begin{aligned} {\text {vol}}M_\gamma = \int _{M_\gamma }\!\! \mathrm{d}{ {\text {vol}}_{M_\gamma }(x)} = \int _M |{\det \mathrm{d}\Phi _\gamma (z)}|\, \mathrm{d}{{\text {vol}}_M(z)}. \end{aligned}$$

Since $1/2 \leqslant |\det \mathrm{d}\Phi _\gamma | \leqslant 2$ for $\gamma$ small enough (depending on *r*), it follows that ${\text {vol}}M/2 \leqslant {\text {vol}}M_\gamma \leqslant 2 {\text {vol}}M$ for the same values of $\gamma$, so that the uniform distribution $P_\gamma$ on $M_\gamma$ in is ${\mathscr {P}}^k_{r/2,2\mathbf{L} ^{*}}(a^*/2,2a^*)$. If we assume that $2\mathbf{L } ^* \leqslant \mathbf{L }$, $f_{\min } \leqslant a^*/2$, and $2a^* \leqslant f_{\max }$ (which we do from now on) then we immediately have $P \in {\mathscr {P}}^k_0$ and $P_\gamma \in {\mathscr {P}}^k_0$, provided that $R_{\text {wfs}}(M_{\gamma }) \geqslant R_{\ell }(M_{\gamma })$. We claim that the latter inequality holds.

Around 0, simple geometrical considerations show that $M_\gamma$ can be viewed as the graph of the function

$$\begin{aligned} \xi _\gamma :{\left\{ \begin{array}{ll} {\mathbf{R }}^d \rightarrow {\mathbf{R }},\\ \displaystyle v \mapsto \sqrt{r^2 - \Vert v\Vert ^2} - r + \gamma ^k \psi \biggl (\dfrac{r}{\gamma }\sqrt{2 -2\sqrt{1 -\Vert v\Vert ^2/r^2}}\biggr ). \end{array}\right. } \end{aligned}$$

Writing $\xi _\gamma (v) = \zeta _\gamma (\Vert v\Vert )$ with $\zeta _\gamma :{\mathbf{R }}\rightarrow {\mathbf{R }}$, a series of computations shows that

$$\begin{aligned} \zeta ''_\gamma (0) = - \dfrac{1}{r} + r \gamma ^{k-2} \psi ''(0). \end{aligned}$$

Setting $c = -\psi ''(0) > 0$ (which depends on *r*) we have, using Lemma 7.4,

$$\begin{aligned} R_{\ell }(M_\gamma ) \leqslant \dfrac{1}{|\zeta ''_\gamma (0)|} = \dfrac{1}{1/r + c r \gamma ^{k-2}} \leqslant r - \dfrac{c r^2 \gamma ^{k-2}}{2} \end{aligned}$$

as soon as $c r^2 \gamma ^{k-2} \leqslant 1$. Now let us turn to the control of $R_{\text {wfs}}(M_\gamma )$. We will show that the distance between any pair of bottleneck points is bounded below by 2*r*. Let $(x,y) \in M_\gamma$ be a pair of bottleneck points. First notice that *x* and *y* cannot lie simultaneously in $B(0,\gamma )$ because $M_\gamma \cap B(0,\gamma )$ can be seen as a graph. If $x,y\in M_\gamma \setminus B(0,\gamma )$, then $d(x,y) = 2 r$ necessarily. If, say, $x \in B(0,\gamma )$ and $y \in M_\gamma \setminus B(0,\gamma )$, then the open segment (*x*, *y*) crosses *M* at a single point $z \in M$. Therefore, we have that $d(x,y) = d(x,z) + d(z,y)$. But now, since [*x*, *y*] is normal to $M_\gamma$ at point *y*, we know that [*z*, *y*] is a diameter of *M* so that $d(z,y)=2r$ and thus $d(x,y) \geqslant 2r$. We have shown that $R_{\text {wfs}}(M_\gamma )\geqslant r\geqslant R_{\ell }(M_\gamma )$ for $\gamma$ small enough and thus $M_\gamma \in {\mathscr {M}}^k_0$ and $P_\gamma \in {\mathscr {P}}^k_0$.

Now, by Lemma 7.3, we have that ${{\,\mathrm{TV}\,}}(P,P_\gamma ) = {\mathscr {H}}^d(M_\gamma \setminus M)/{{\text {vol}}M_\gamma }\leqslant C \gamma ^d$ for some constant *C* depending on *r*. Applying now Le Cam’s Lemma 7.2 and noting that $R(M) - R(M_\gamma ) \geqslant cr^2 \gamma ^{k-2}$, we obtain

$$\begin{aligned} \inf _{{\widehat{R}}} \sup _{P \in {\mathscr {P}}^k_0} {\mathbf{E }}_{P^{\otimes n}}[|{\widehat{R}} - R|] \geqslant \dfrac{cr^2 \gamma ^{k-2}}{2} (1 - C \gamma ^d)^n. \end{aligned}$$

Setting $\gamma = 1/(Cn)^{1/d}$, we know that for *n* large enough (depending on *r*), we have

$$\begin{aligned} \inf _{{\widehat{R}}} \sup _{P \in {\mathscr {P}}^k_0} {\mathbf{E }}_{P^{\otimes n}}[|{\widehat{R}} - R|] \geqslant \dfrac{cr^2 (Cn)^{-(k-2)/d}}{8} . \end{aligned}$$

Set *r* to be equal to $2R_{\min }$ and the first statement of Theorem 7.1 follows.

*Step 2: The case of* ${\mathscr {P}}^k_\alpha$. We next turn to the second part of the theorem. We fix $\alpha > 0$ and construct a manifold $M \in {\mathfrak {C}}^k$ as follows. We consider the two parallel disks $B(0,2r) \subseteq {\mathbf{R }}^d \subseteq {\mathbf{R }}^{d+1}$ and $B(2r e_{d+1},2r) \subseteq 2r e_{d+1} + {\mathbf{R }}^d \subseteq {\mathbf{R }}^{d+1}$, with $r \geqslant 2R_{\min }$, and link them together so that *M* satisfies the following:

- *M* is a smooth submanifold of ${\mathbf{R }}^{d+1}$,
- *M* has reach *r* and $(0,2r e_{d+1})$ is a reach attaining pair,
- $$R_{\ell }(M) \geqslant r + \alpha $$.

**Fig. 4:**
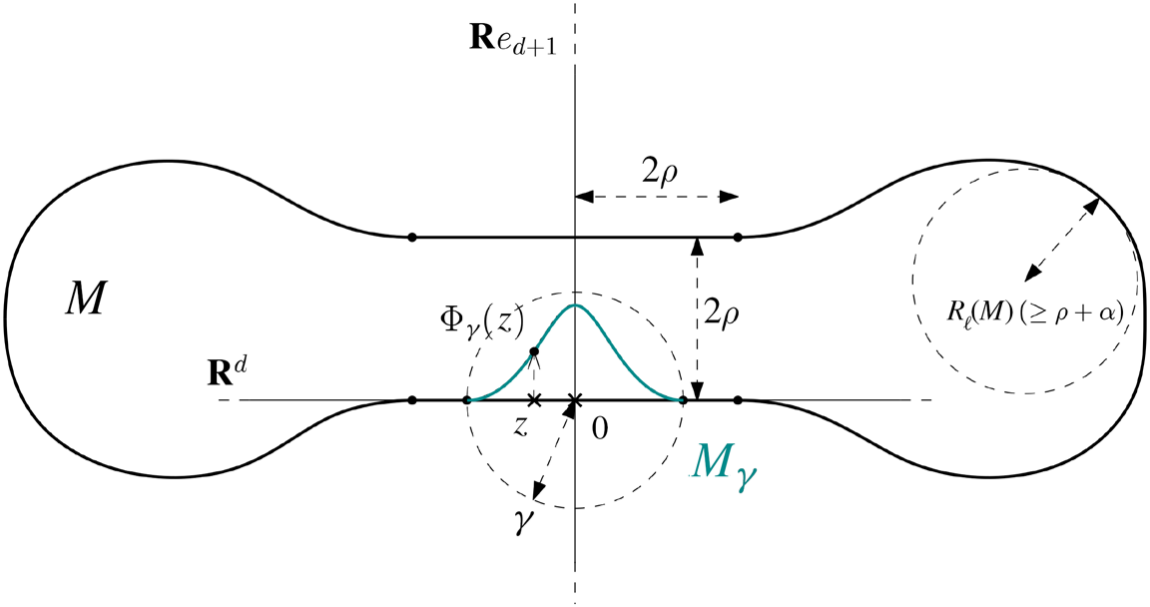
The submanifolds *M* and $M_\gamma$ used in the second part of the proof of the lower bound

See Fig. 4 for a schematic notion of such *M*, visualized with $d=1$.

Furthermore, we know that there exists $\mathbf{L } ^*$ (depending on *r* and $\alpha$) such that $M \in {\mathfrak {C}}^k_{r, \mathbf{L } ^*}$ and $P \in {\mathscr {P}}^k_{r,\mathbf{L } ^*}(a^*, a^*)$ where $a^* = 1/{{\text {vol}}M}$ and where *P* is the uniform probability over *M*. We again consider the map

$$\begin{aligned} \Phi _\gamma :{\left\{ \begin{array}{ll} {\mathbf{R }}^{d+1} \rightarrow {\mathbf{R }}^{d+1}, \\ z \mapsto z + \gamma ^k \Psi (z/\gamma ) e_{d+1}. \end{array}\right. } \end{aligned}$$

Similarly to the first part of the theorem, for $\gamma$ small enough (depending on $\alpha$ and *r*), we know that $M_\gamma = \Phi _\gamma (M)$ is a smooth submanifold in ${\mathfrak {C}}^k_{r/2, 2\mathbf{L } ^*}$ and that the uniform distribution $P_\gamma$ over $M_\gamma$ lies in $\mathscr {P}^k_{r/2,2\mathbf{L} ^*}(a^*/2,2a^*)$. Again, assuming that $\mathbf{L } \geqslant 2\mathbf{L } ^*$, $f_{\min } \leqslant a^*/2$, and $f_{\max } \geqslant 2a^*$, we have that $P \in {\mathscr {P}}^k_\alpha$ and, furthermore, that $P_\gamma \in {\mathscr {P}}^k_\alpha$, provided $R_{\ell }(M_\gamma ) \geqslant R_{\text {wfs}}(M_\gamma ) + \alpha$. We claim that the latter inequality holds.

Since $\Psi$ is maximal at 0, we know that $(\gamma ^k \psi (0) e_{d+1}, 2r e_{d+1})$ is still a bottleneck pair, and thus $R_{\text {wfs}}(M_\gamma ) \leqslant r - c \gamma ^k$ where we set $c = -2\psi (0)$ (depending on $\alpha$ and *r*). For the curvature, notice that it is unchanged outside of $B(0,\gamma )$ and that $M_\gamma$ is just the graph of $v \mapsto \gamma ^k \Psi (v/\gamma )$ within this ball. Using Lemma 7.4, we thus have $R_{\ell }(M_{\gamma }) \geqslant \min {\lbrace r + \alpha , (C\gamma ^{k-2})^{-1}\rbrace }$, with *C* depending on $\alpha$ and *r*, so that $R_{\ell }(M_\gamma ) \geqslant R_{\text {wfs}}(M_\gamma ) + \alpha$ for $\gamma$ small enough (depending on $\alpha$ and *r*), and therefore $M_\gamma \in {\mathscr {M}}^k_\alpha$ and $P_\gamma \in {\mathscr {P}}^k_\alpha$.

Using Lemma 7.3, we have that ${{\,\mathrm{TV}\,}}(P,P_\gamma ) = {\mathscr {H}}^d(M_\gamma \setminus M)/{{\text {vol}}M_\gamma } \leqslant \delta \gamma ^d$ for some constant $\delta$ depending on *r*. Applying now Le Cam’s Lemma 7.2 and noticing that $R(M) - R(M_\gamma ) \geqslant c \gamma ^{k}$, we get

$$\begin{aligned} \inf _{{\widehat{R}}} \sup _{P \in {\mathscr {P}}^k_0} {\mathbf{E }}_{P^{\otimes n}}[|{\widehat{R}} - R|] \geqslant \dfrac{c \gamma ^{k}}{2} (1 - \delta \gamma ^d)^n. \end{aligned}$$

Setting $\gamma = 1/(\delta n)^{1/d}$, we know that for *n* large enough (depending on *r* and $\alpha$), we have

$$\begin{aligned} \inf _{{\widehat{R}}} \sup _{P \in {\mathscr {P}}^k_0} {\mathbf{E }}_{P^{\otimes n}}[|{\widehat{R}} - R|] \geqslant \dfrac{c (\delta n)^{-k/d}}{8} . \end{aligned}$$

Setting $r = 2R_{\min }$ yields the result, completing the proof of Theorem 7.1. $\square$

## References

[1] Aamari E, Kim J, Chazal F, Michel B, Rinaldo A, Wasserman L (2019). Estimating the reach of a manifold. Electron. J. Stat..

[2] Aamari E, Levrard C (2018). Stability and minimax optimality of tangential Delaunay complexes for manifold reconstruction. Discrete Comput. Geom..

[3] Aamari E, Levrard C (2019). Nonasymptotic rates for manifold, tangent space and curvature estimation. Ann. Stat..

[4] Almgren F (1986). Optimal isoperimetric inequalities. Indiana Univ. Math. J..

[5] Attali D, Lieutier A (2015). Geometry-driven collapses for converting a Čech complex into a triangulation of a nicely triangulable shape. Discrete Comput. Geom..

[6] Attali D, Lieutier A, Salinas D (2013). Vietoris-Rips complexes also provide topologically correct reconstructions of sampled shapes. Comput. Geom..

[7] Balakrishnan, S., Rinaldo, A., Sheehy, D., Singh, A., Wasserman, L.: Minimax rates for homology inference. In: 15th International Conference on Artificial Intelligence and Statistics (Los Cancajos 2012). Proceedings of Machine Learning Research, vol. 22, pp. 64–72 (2012)

[8] Boissonnat J-D, Lieutier A, Wintraecken M (2019). The reach, metric distortion, geodesic convexity and the variation of tangent spaces. J. Appl. Comput. Topol..

[9] do Carmo MP (1992). Riemannian Geometry. Mathematics: Theory & Applications.

[10] Divol, V.: Minimax adaptive estimation in manifold inference (2020). arXiv:2001.04896

[11] Efron B, Tibshirani RJ (1993). An introduction to the bootstrap. Monographs on Statistics and Applied Probability.

[12] Federer H (1959). Curvature measures. Trans. Am. Math. Soc..

[13] Genovese CR, Perone-Pacifico M, Verdinelli I, Wasserman L (2012). Minimax manifold estimation. J. Mach. Learn. Res..

[14] Grove, K.: Critical point theory for distance functions. In: Differential Geometry: Riemannian Geometry (Los Angeles 1990). Proceedings of Symposia on Pure Mathematics, vol. 54, part 3, pp. 357–385. American Mathematical Society, Providence (1993)

[15] Kim J, Rinaldo A, Wasserman L (2019). Minimax rates for estimating the dimension of a manifold. J. Comput. Geom..

[16] Lytchak A (2004). On the geometry of subsets of positive reach. Manuscr. Math..

[17] Niyogi P, Smale S, Weinberger S (2008). Finding the homology of submanifolds with high confidence from random samples. Discrete Comput. Geom..

[18] Yu B (1997). Assouad, Fano, and Le Cam. Festschrift for Lucien Le Cam.

